# Dual-function hemoglobin-encapsulating ZIF-8 nanoparticles: Oxygen transport capability and carbonic anhydrase-like activity

**DOI:** 10.1016/j.mtbio.2025.102406

**Published:** 2025-10-10

**Authors:** Ana María Pablo-Sainz-Ezquerra, Marta Rubio-Huertas, Ege Tini Tunca, Peter Waaben Thulstrup, Leticia Hosta-Rigau

**Affiliations:** a Department of Health Technology, Technical University of Denmark, Nils Koppels Allé, Building 423, 2800, Kgs. Lyngby, Denmark; b Department of Chemistry, University of Copenhagen, Universitetsparken 5, 2100, Copenhagen, Denmark

**Keywords:** Blood substitutes, Carbonic anhydrase, Hemoglobin-based oxygen carriers, Nanozymes, Metal-organic frameworks, ZIF-8

## Abstract

Hemoglobin-based oxygen carriers (HBOCs) have emerged as a promising alternative to red blood cell (RBC) transfusions, addressing key limitations such as cold storage requirements, restricted availability of universal donor blood, and the need for cross-matching, which collectively hinder their immediate use in emergency settings. Despite their potential, existing HBOCs primarily focus on oxygen delivery while overlooking a crucial physiological function of native RBCs: carbon dioxide (CO_2_) transport, which is essential not only for gas exchange but also for acid-base homeostasis.

In this study, we explore the potential of our previously developed hemoglobin (Hb)-loaded zeolitic imidazole framework-8 nanoparticles (Hb@ZIF-8 NPs) as a dual-function RBC substitute capable of both oxygen delivery and CO_2_ transport. ZIF-8, a metal-organic framework (MOF), was selected due to its high porosity, biocompatibility, and structural resemblance to the catalytic center of carbonic anhydrase (CA), the native enzyme responsible for catalyzing the reversible hydration of CO_2_ within RBCs. Our results show that, similarly to CA, Hb@ZIF-8 NPs synthesized with polyethylene glycol (PEG) as a seeding agent and varying Hb concentrations can catalyze the hydrolysis of *p*-nitrophenyl acetate (*p*-NPA) into *p*-nitrophenol (*p*-NP), which can be detected spectrophotometrically. Notably, enzyme kinetics analysis reveals that Hb@ZIF-8 NPs follow Michaelis-Menten kinetics, with coefficients of determination (R^2^) exceeding 0.99 in most cases, indicating strong adherence to enzyme behavior. The maximum reaction velocity (V_max_) decreased with PEG and increasing Hb content, likely due to reduced availability of the MOF's metal ion, which acts as the active catalytic sites. However, the Michaelis-Menten constant (K_m_) suggests that the presence of PEG and Hb enhances substrate affinity, possibly through interactions with *p*-NPA. The catalytic efficiency (K_eff_) was higher in Hb@ZIF-8 NPs with greater Hb content, suggesting improved enzymatic activity alongside oxygen transport.

These findings establish Hb@ZIF-8 NPs as HBOCs also capable of mimicking CA esterase activity, reinforcing their dual role in oxygen and CO_2_ transport. This integration of oxygen-carrying capacity with CA-like functionality represents a significant advancement in the development of artificial RBCs, with potential clinical implications for improving systemic oxygenation and preventing acidosis in transfusion-limited or emergency settings.

## Graphical abstract

**Figure:**
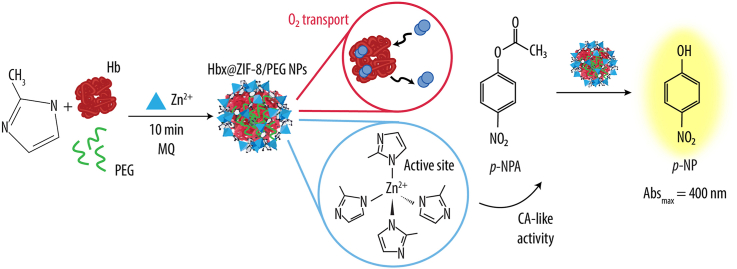

## 1 Introduction

With 5.6 million lives lost annually to trauma, and nearly half of these fatalities attributed to uncontrolled bleeding, timely administration of red blood cells (RBCs) is essential for restoring oxygen transport and improving survival rates [1,2]. However, several challenges hinder the immediate use of donor RBCs in emergency settings. The need for cold storage, the limited availability of universal donor blood (O-negative), and the requirement for cross-matching contribute to delays in transfusion, restricting its use primarily to hospital settings [[3], [4], [5]]. Additionally, the 42-day shelf life of stored RBCs complicates efforts to maintain sufficient reserves for mass casualty events such as natural disasters or terrorist attacks. These logistical constraints, combined with periodic blood shortages and concerns over donor availability, highlight the urgent need to develop innovative oxygen-carrying solutions to sustain life in the timeframe between acute blood loss and the eventual transfusion of RBCs.

Hemoglobin-based oxygen carriers (HBOCs) represent a promising alternative to traditional blood transfusions, offering the advantages of universal compatibility, long-term storage, and rapid oxygen delivery [[3], [4], [5], [6]]. Unlike donor RBCs, HBOCs do not require blood typing and can potentially be stored as freeze-dried powders, enhancing their accessibility in emergency and battlefield settings. However, despite their potential, the clinical translation of HBOCs has been hindered by significant challenges. The primary concern stems from the toxicity of free hemoglobin (Hb) once it is removed from the protective RBC membrane, which can extravasate through the vascular endothelium and into the underlying smooth muscle tissue, leading to vasoconstriction, systemic hypertension, and oxidative tissue injury. [3,5,7] To stabilize Hb and prevent its extravasation, various strategies (including polymerization [8], conjugation to poly(ethylene glycol) (PEG) and encapsulation within liposomes) [9] have been explored. However, chemical modifications such as polymerization and conjugation, while stabilizing Hb, often come at the cost of reduced oxygen-binding efficiency [10]. In contrast, liposomal encapsulation preserves oxygen transport properties but suffers from low encapsulation efficiency (EE), limiting scalability and commercial viability [11].

Furthermore, even if the current challenges in artificial oxygen carriers were overcome, it is important to recognize that most reported systems focus solely on oxygen transport. However, in addition to delivering oxygen, biological RBCs exhibit antioxidant functions and also play a crucial role in carbon dioxide (CO_2_) transport. While some advanced encapsulation strategies have incorporated Hb alongside reducing agents and antioxidant compounds, such as enzymes or nanozymes, a critical yet often overlooked function of natural RBCs is the transport of CO_2_ from tissues to the lungs for excretion. The carbonic acid/bicarbonate buffer system plays a key role in maintaining acid-base balance in the blood, and excessive CO_2_ accumulation leads to increased plasma proton (H^+^) concentrations, which can result in acidosis, central nervous system dysfunction, coma, and even death. Since CO_2_ has low solubility in water, only about 5 % exists as dissolved gas, while the majority is converted into bicarbonate (HCO_3_^−^), a reaction that is too slow to meet physiological demands without enzymatic catalysis. In biological RBCs, this process is efficiently facilitated by carbonic anhydrase (CA), enabling sufficient CO_2_ transport and excretion [12,13]. A solitary example has attempted to address CO_2_ transport by co-polymerizing Hb with the CA enzyme [14]. Notably, the *in vivo* results showed that polymerized Hb and CA were able to lower the elevated tissue CO_2_ level in a hemorrhagic shock rat model more effectively than native RBC [12]. Despite this encouraging result, the use of natural enzymes in HBOCs presents important limitations: enzymes are expensive, difficult to produce at scale, and prone to instability in physiological media [15]. Moreover, their chemical modification to allow incorporation into polymerized Hb may lead to altered functionality.

Recently, we developed a simple, rapid, and scalable method for synthesizing HBOCs with a high content of functional Hb [16]. This approach utilizes zeolitic imidazolate framework-8 (ZIF-8), a well-characterized metal-organic framework (MOF), as an encapsulation matrix [17]. ZIF-8 nanoparticles (NPs) are particularly well-suited for this application due to their high porosity, which facilitates oxygen transport, and excellent biocompatibility, making them an ideal platform for Hb stabilization and delivery [16,18]. While other MOFs such as UiO-66 [19] or MIL100 [20] have also shown promise for protein encapsulation, ZIF-8 NPs uniquely mimic CA activity due to their structural resemblance to the enzyme's active center, namely a zinc (Zn^2+^) metallic center coordinated to imidazolate [21]. CA's activity originates from its Zn^2+^-containing active site, in which three histidine moieties coordinate a Zn^2+^ ion leaving a vacant space for the association of a water molecule. This enables ZIF-8 to function not only as a stabilizing and oxygen-permeable host for Hb but also as a catalyst capable of accelerating CO_2_ hydration, thereby addressing two critical functions of native RBCs within a single platform.

**Scheme 1:**
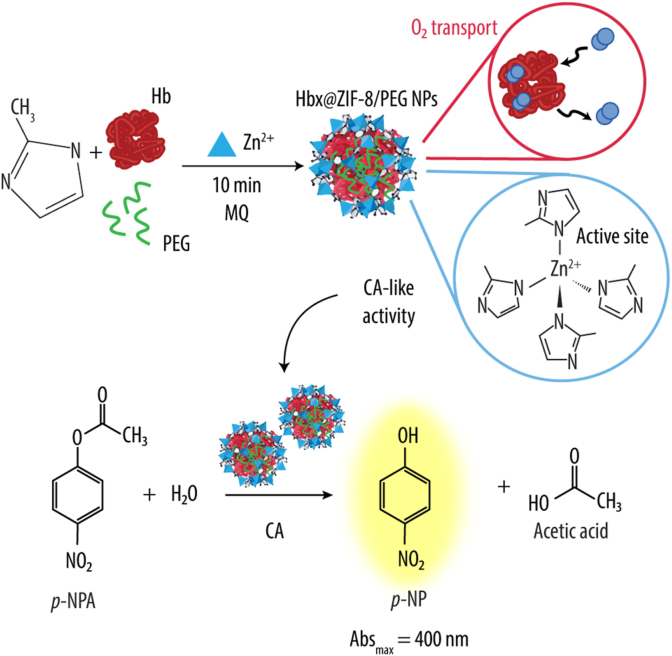
Schematic illustration of the one-pot synthesis of hemoglobin (Hb)-loaded ZIF-8 nanoparticles (Hbx@ZIF-8/PEG NPs) using polyethylene glycol (PEG) as a capping agent. The synthesis involves mixing Hb, 2-methylimidazole (HmIm), PEG, zinc ions (Zn^2+^) and Milli-Q (MQ) water for 10 min. The resulting Hbx@ZIF-8/PEG NPs enable oxygen (O_2_) transport via encapsulated Hb and exhibit carbonic anhydrase (CA)-like activity, due to the structural similarity between the CA active site and the ZIF-8 framework. This CA-mimetic function is demonstrated by the ability of both CA and Hbx@ZIF-8/PEG NPs to catalyze the hydrolysis of *p*-nitrophenyl acetate (*p*-NPA) into *p*-nitrophenol (*p*-NP) and acetic acid. The reaction is monitored by spectrophotometry, with *p*-NP showing a characteristic absorbance maximum (Abs_max_) at 400 nm.

Thus, this work extends our previous findings on Hb-loaded ZIF-8 NPs fabricated using PEG as a nucleation agent (i.e., Hb@ZIF-8/PEG NPs) by demonstrating not only their oxygen-binding and release properties but also for their capacity to mimic CA esterase activity (Scheme 1).

## 2 Materials and methods

### 2.1 Materials

Bovine blood with citrate was purchased from SSI Diagnostica A/S (Hillerød, DK). Zinc nitrate hexahydrate (Zn(NO_3_)_2_·6H_2_O), 2-methylimidazole (HmIm), ethylenediaminetetraacetic acid (EDTA) solution, carbonic anhydrase (CA) from bovine erythrocytes lyophilized powder (≥2000 W-A units/mg protein), tris(hydroxymethyl) aminomethane (TRIS), bis(2-hydroxyethyl)aminotris(hydroxymethyl)methane (BIS-TRIS), sodium chloride (NaCl), polyethylene glycol (PEG,-OH terminated, M_W_ 6000 Da), hydrogen peroxide (H_2_O_2_) solution (30 % Suprapur), Iron (Fe) Standard for ICP: TraceCERT®, 1 g L^−1^ Fe in nitric acid (nominal concentration), *p*-nitrophenyl acetate (*p*-NPA), hemoglobin (Hb) from bovine blood in lyophilized powder, sodium dithionite (SDT), calcein-acetoxymethyl (calcein-AM) and phosphate buffer saline (PBS) in tablets were obtained from Merck Life Sciences A/S (Søborg, DK). Methanol (MeOH) (≥99.9 %) dimethyl sulfoxide (DMSO) and nitric acid (HNO_3_) 68 %, were acquired from VWR International (Søborg, DK). Pierce™ Bicinchoninic acid (BCA) Protein Assay Kit was purchased from Thermo Fisher Scientific (Roskilde, DK).

All chemicals were used without further modification, except for CA, which was dissolved in a solution of TRIS (20 mM, pH 7.4) and NaCl (150 mM), and stored in aliquots at −20 °C and calcein-AM, which was dissolved (5 mM in DMSO) and stored in aliquots at −20 °C.

Ultrapure water (Milli-Q (MQ), Gradient A 10 system, TOC <4 ppb, resistance 18 MΩ cm, EMD Millipore) was used to prepare the solutions.

### 2.2 Hb extraction from bovine blood

Hb was extracted from bovine blood by hypotonic hemolysis with modification to a reported protocol [22]. In brief, the blood was washed (3 × , 2000*g*, 20 min, 4 °C) with a NaCl solution (0.9 %, 1:4 v/v) using a high-speed centrifuge (SL16R centrifuge, Thermo Scientific, Hvidovre, DK). The plasma was removed and the pellet containing the RBCs was diluted twofold in PBS. The RBCs were washed with 20 mM PBS (4 × , 10 000*g*, 5 min, 4 °C). To lyse the RBCs, the obtained pellet was thoroughly mixed with three volumes of MQ. The resulting mixture was stored overnight at 4 °C in a stoppered flask for Hb separation. Hb was separated from cell debris by vacuum filtration. Aliquots of free Hb (2 mL) were prepared and stored at −80 °C for future use.

### 2.3 NP synthesis

#### 2.3.1 ZIF-8 NPs

ZIF-8 NPs were synthesized with slight modifications to previously reported one-pot methods [16,23]. A schematic of the synthesis procedure is shown in Fig. S1A (Supporting Information). The reaction was carried out at room temperature (RT) using MeOH as the solvent. Briefly, Zn(NO_3_)_2_·6H_2_O (8 mL, 100 mM in MeOH) was added to HmIm (8 mL, 400 mM in MeOH) in a 40 mL glass vial, maintaining a Zn^2+^:HmIm molar ratio of 1:4. The mixture was stirred at 800 rpm for 10 min using a magnetic stirrer and then left to age undisturbed for 24 h at RT. The resulting ZIF-8 NPs were collected and washed by centrifugation (3 × , 12 000*g*, 10 min, 4 °C, MeOH) and subsequently activated by overnight drying in a vacuum oven at 60 °C. The resulting powder containing ZIF-8 NPs was redispersed in MQ by ultrasonication for 30 min to a final concentration of 10 mg mL^−1^ and stored for at 4 °C for future use (2 weeks maximum).

#### 2.3.2 ZIF-8/PEG NPs

ZIF-8/PEG NPs were synthesized by adapting previously described one-pot procedures [16,23]. A schematic of the synthesis procedure is shown in Fig. S1B (Supporting Information). The synthesis was conducted at RT using MQ as the solvent. In a 40 mL vial, MQ (5.013 mL) was first added, followed sequentially by PEG (2.665 mL, 1200 mg mL^−1^), HmIm (320 μL, 2 M) and Zn(NO_3_)_2_·6H_2_O (8 mL, 20 mM). The final concentrations were of 200 mg mL^−1^ (PEG), 40 mM (HmIm) and 10 mM (Zn^2+^) maintaining a Zn^2+^:HmIm molar ratio of 1:4. The reaction was stirred for 10 min at 800 rpm using a magnetic stirrer. The resulting ZIF-8/PEG NPs were collected and washed by centrifugation (3 × , 12 000*g*, 10 min, 4 °C, MQ) and activated by overnight drying in a vacuum oven at 60 °C. The resulting dried powder was redispersed in MQ using ultrasonication for 30 min to a final concentration of 10 mg mL^−1^ and stored at 4 °C until further use (for 2 weeks maximum).

#### 2.3.3 Hbx@ZIF-8/PEG NPs (x = 0.6, 3, 6, 13, 25)

Hbx@ZIF-8/PEG NPs were synthesized following a previously reported one-pot method developed by our group [16]. A schematic of the synthesis procedure is shown in Fig. S1C (Supporting Information). Briefly, PEG, Hb, HmIm and Zn(NO_3_)_2_·6H_2_O were added sequentially to MQ, in the order listed, to obtain final concentrations of 200 mg mL^−1^ (PEG), 40 mM (HmIm), and 10 mM (Zn^2+^). Hb was added (11.2, 55.9, 111.9, 223.7 and 447.4 μL, 112.9 mg mL^−1^, MQ) to obtain final concentrations of 0.6, 3.2, 6.3, 12.6 and 25.2 mg mL^−1^ yielding Hb0.6@ZIF-8/PEG NPs, Hb3@ZIF-8/PEG NPs, Hb6@ZIF-8/PEG NPs, Hb13@ZIF-8/PEG NPs and Hb25@ZIF-8/PEG NPs, respectively. The solutions were stirred at 800 rpm for 10 min using a magnetic stirrer. The resulting Hbx@ZIF-8/PEG NPs were collected and washed by centrifugation (3 × , 12 000*g*, 10 min, 4 °C, MQ). The final Hbx@ZIF-8/PEG NPs were redispersed (2 mL, MQ) and stored at 4 °C until further use (for 2 weeks maximum).

### 2.4 NPs physicochemical characterization

At least two independent batches were prepared for each type of NP.

#### 2.4.1 Surface charge

The zeta-(ζ)-potential of diluted ZIF-8, ZIF-8/PEG and Hbx@ZIF-8/PEG NPs (1:40 v/v in MQ) was measured using a Zetasizer Nano-ZS (Malvern Panalytical Ltd., Malvern, UK).

#### 2.4.2 Hb quantification

##### 2.4.2.1 Inductively coupled plasma mass spectrometry (ICP-MS)

ICP-MS was used to quantify the iron (Fe) content in Hb extracted from bovine RBCs and in Hbx@ZIF-8/PEG NPs. Due to the low Fe content of some samples, different volumes were used for digestion: Hb0.6@ZIF-8/PEG NPs (500 μL, 1.3 mg mL^−1^), Hb3@ZIF-8/PEG NPs (100 μL, 3.7 mg mL^−1^), Hb6@ZIF-8/PEG NPs (50 μL, 8.1 mg mL^−1^), Hb13@ZIF-8/PEG NPs (50 μL, 14.6 mg mL^−1^), Hb25@ZIF-8/PEG (50 μL, 27.7 mg mL^−1^). Due to the high content of Fe, the Hb stock was diluted 20 times in MQ and 50 μL of the dilution was used for digestion. Each sample was digested with 100 μL of 30 % H_2_O_2_ and 50 μL of 65 % HNO_3_. Additionally, for the Hb6@ZIF-8/PEG, Hb13@ZIF-8/PEG, Hb25@ZIF-8/PEG NPs and Hb stock, 50 μL of MQ water was added. Digestion was ensured by incubating the samples in a thermoshaker (AccuTherm Microtube Shaking Incubator, Labnet International Inc., Edison, US) at 60 °C and 300 rpm for 90 min. After digestion, the samples were diluted with a 2 % (v/v) HNO_3_ aqueous solution (prepared with MQ water) to achieve a final Fe concentration of 100–160 ppb. Blanks were prepared similarly, using MQ in place of the sample volume, and a standard curve was generated using a commercial Fe standard treated in the same way. All samples were prepared in triplicate, and each replicate was measured seven times.

##### 2.4.2.2 SLS-based UV–vis

The Hb concentration was also determined via a UV–vis-based SLS assay, following a method previously developed by our group [22]. Briefly, Hbx@ZIF-8/PEG NPs (100 μL, 0.9–28 mg mL^−1^) were disassembled using EDTA (100 μL, 50 mM). Different dilutions of each disassembled sample were prepared (2x, 4x, 8x, 16x and 32x, MQ) in a transparent 96-well plate (Corning™ Costar™, Cell Culture Treated, Flat-Bottom). Then, 10 μL from each well was transferred to a new plate and SLS (200 μL, 0.6 mg mL^−1^) was added to each well. The plate was covered with aluminum foil and stirred for 5 min using a well-plate stirrer. The absorbance (Abs) at 539 nm was measured using a plate reader (Tecan Spark, Tecan Group Ltd., Männedorf, CH). A standard curve was generated using a Hb stock solution characterized by ICP-MS.

#### 2.4.3 NPs quantification

The concentration of ZIF-8, ZIF-8/PEG, and Hb_x_@ZIF-8/PEG NPs (x = 0.6, 3, 6, 13, 25) was determined gravimetrically by freeze-drying a known volume of each sample and measuring the resulting dry mass:

(1) NPs (mg mL^−1^) = (Mass of freeze-dried NPs)/(Freeze dried volume)

#### 2.4.4 Hb content, EE and loading content (LC)

Hb content, EE and LC were calculated using the following formulas:

(2) Hb content (%) = (Hb entrapped in the Hbx@ZIF-8/PEG NPs determined through ICP-MS)/(Concentration of freeze-dried Hbx@ZIF-8/PEG NPs) × 100

(3) EE (%) = (Hb entrapped in the Hbx@ZIF-8/PEG NPs determined through SLS)/(Initial added Hb) × 100

(4) LC (%) = (Hb entrapped in the Hbx@ZIF-8/PEG NPs determined through SLS)/(Concentration of freeze-dried Hbx@ZIF-8/PEG NPs) × 100

#### 2.4.5 Zn quantification

Zn content in the Hb solutions used for synthesis, as well as in ZIF-8, ZIF-8/PEG, and Hbx@ZIF-8/PEG NPs, was quantified using ICP-MS. For sample digestion, Hb0.6@ZIF-8/PEG NPs (500 μL, 1.3 mg mL^−1^), Hb3@ZIF-8/PEG NPs (100 μL, 3.7 mg mL^−1^), Hb6@ZIF-8/PEG NPs (50 μL, 8.1 mg mL^−1^), Hb13@ZIF-8/PEG NPs (50 μL, 14.6 mg mL^−1^), Hb25@ZIF-8/PEG (50 μL, 27.7 mg mL^−1^) and Hb stock (50 μL, 20x dilution) were mixed with commercial H_2_O_2_ (100 μL, 30 %) and HNO_3_ (50 μL, 65 %). Additionally, for the Hb6@ZIF-8/PEG, Hb13@ZIF-8/PEG, Hb25@ZIF-8/PEG and Hb stock samples, 50 μL of MQ water was added. The samples were incubated (60 °C, 300 rpm, 90 min) in a thermoshaker (AccuTherm Microtube Shaking Incubator, Labnet International Inc., Edison, US) to ensure complete digestion. Samples were then diluted with HNO_3_ (2 % v/v, MQ) to achieve final Zn concentrations between 30 and 40 ppb. Blanks were prepared by replacing the equivalent sample volume with MQ water and a standard curve was generated using a commercial Zn standard treated in the same way. All samples were prepared in triplicate, and each replicate was measured seven times.

#### 2.4.6 Yield

The reaction yield was calculated based on Zn quantification using the following equation:

(5) Yield (%) = (Zn concentration determined by ICP-MS)/(Theoretical Zn concentration in the reaction mixture) × 100

#### 2.4.7 CA quantification

CA concentration was determined using a BCA protein assay kit, following the manufacturer's instructions [24]. Briefly, 25 μL of each CA stock aliquot was loaded in triplicate into transparent 96 well-plates (Corning™ Costar™, Cell Culture Treated, Flat-Bottom). A bovine serum albumin (BSA) standard (2.0 mg mL^−1^) included in the kit was used to construct a standard curve. Next, 200 μL of BCA working reagent (prepared at a 50:1 ratio of reagent A to reagent B) was added to each well. The plate was shaken for 30 s in a plate shaker (PMS-1000i, Grant Instruments, Amsterdam NL), covered with aluminum foil, and incubated for 30 min at 37 °C. The Abs at 562 nm was measured using a plate reader (Tecan Spark, Tecan Group Ltd., Männedorf, CH).

#### 2.4.8 Scanning electron microscopy (SEM)

SEM analysis was conducted using a Quanta FEG 250 Analytical Environmental SEM (FEI Company, Hillsboro, US). Diluted suspensions of ZIF-8, ZIF-8/PEG, and Hbx@ZIF-8/PEG NPs in MQ were drop-casted onto glass slides mounted on top of adhesive carbon tabs. After drying, the samples were sputter-coated with gold for 15 s at 20 mA using a Quorum Q150T system (Quorum Technologies Ltd., UK). Images were captured using an Everhart-Thornley detector at an accelerating voltage of 10–20 kV. The size was calculated by measuring 150 NPs using ImageJ software and fitting the data to a lognormal distribution in OriginPro2024. The diameter of the NPs was the center of the distribution (xc) and the polydispersity index (PDI) was obtained from the log standard deviation (ɷ).

#### 2.4.9 Transmission electron microscopy (TEM)

TEM imaging was performed using a Tecnai G2 T20 Microscope (FEI Company, Hillsboro, US). Diluted suspensions of ZIF-8, ZIF-8/PEG, and Hbx@ZIF-8/PEG NPs in MQ were drop-casted onto lacey carbon film-coated copper TEM grids (Agar Scientific Ltd., Stansted Essex, UK). Images were acquired at an accelerating voltage of 200 kV.

#### 2.4.10 Powder X-ray diffraction (PXRD)

A Malvern Panalytical Aeris Research benchtop powder diffractometer (Malvern Instruments Ltd., Malvern, UK) equipped with a Cu Kα X-ray source (40 kV, 15 mA, λ = 1.5406 Å) was used. Diffraction patterns were collected over a 2θ range of 5–35° 2θ, with a step size of 0.011° and a time per step of 109.65 s. The instrument was configured with a 1/4° divergence slit, a nickel beta-filter, 0.04 rad Soller slits, a 9 mm anti-scatter slit, and a low beam knife. Samples were loaded onto zero-background holders made from single crystal silicon (PW1817/32, Panalytical) due to the limited amount of sample available. The sample stage was rotated at 0.5 rev s^−1^. To ease preparation, a few drops of EtOH were used to deposit the powder onto the holder's surface. Collected diffraction patterns were analyzed using HighScore Plus (Malvern Panalytical) software and the peak positions were compared to simulated ZIF-8 polymorph diffraction patterns from the Cambridge Structural Database of the Cambridge Crystallographic Data Centre, with deposition numbers 864312 and 1032088 [25,26]. The crystal size and the crystallinity index were calculated using OriginPro2024 Software.

### 2.5 Catalytic activity

Two independent batches were prepared for each type of NP.

#### 2.5.1 CA esterase activity

CA solutions (20 μL, 50, 100, 500 and 5000 μg mL^−1^ in TRIS) were added in triplicate to a transparent 96-well plate (Corning™ Costar™, Cell Culture Treated, Flat-Bottom). To each well, *p*-NPA (20 μL, 2 mM in EtOH) and TRIS buffer (25 mM, pH 7.4) or MQ were added to reach a final reaction volume of 200 μL. The resulting final concentrations were 5, 10, 50 and 500 μg mL^−1^ for CA, 0.2 mM for *p*-NPA, and 20 mM for TRIS. To correct for intrinsic absorbance, a background control was prepared by replacing *p*-NPA with an equal volume of EtOH. A reference control included only *p*-NPA (20 μL, 2 mM in EtOH) and TRIS buffer (25 mM, pH 7.4) or MQ. The reaction mixtures were incubated at RT for 90 min without shaking. The Abs was recorded at 400 nm over time using a plate reader (Tecan Spark, Tecan Group Ltd., Männedorf, CH). Additional Abs measurements were taken at 318 and 348 nm after 90 min of reaction.

#### 2.5.2 Hb activity

Hb solutions (20 μL, 50, 100 and 500 μg mL^−1^ in TRIS or MQ) were added in triplicate to a transparent 96 well-plate (Corning™ Costar™, Cell Culture Treated, Flat-Bottom). To each well, *p*-NPA (20 μL, 2 mM in EtOH) and TRIS buffer (25 mM, pH 7.4) or MQ were added to reach a final reaction volume of 200 μL. The resulting final concentrations were 5, 10 and 50 μg mL^−1^ for Hb, 0.2 mM for *p*-NPA and 20 mM for TRIS. To correct for intrinsic absorbance, a background control was prepared by replacing *p*-NPA with an equal volume of EtOH. A reference control included only *p*-NPA (20 μL, 2 mM in EtOH) and MQ or TRIS (20 mM). The reaction mixtures were incubated at RT for 90 min without shaking and the Abs at 400 nm was recorded over time using a plate reader (Tecan Spark, Tecan Group Ltd., Männedorf, CH).

#### 2.5.3 NPs esterase activity

ZIF-8, ZIF-8/PEG and Hbx@ZIF-8/PEG NPs (x = 0.6, 3, 6, 13, 25) suspensions (20 μL, 0–5 mg mL^−1^ in MQ) were added in triplicate to a transparent 96 well-plate (Corning™ Costar™, Cell Culture Treated, Flat-Bottom). To each well, *p*-NPA (20 μL, 2 mM in EtOH) and MQ were added to reach a final reaction volume of 200 μL. The resulting final concentrations were 10, 50, 100, 250 and 500 μg mL^−1^ for the NPs and 0.2 mM for *p*-NPA. To correct for intrinsic absorbance, a background control was prepared by replacing *p*-NPA with an equal volume of EtOH. A reference control included only *p*-NPA (20 μL, 2 mM in EtOH) and MQ. The reaction mixtures were incubated at RT for 90 min without shaking. Abs was recorded over time at 400 nm using a plate reader (Tecan Spark, Tecan Group Ltd., Männedorf, CH).

#### 2.5.4 Michaelis-Menten parameters

ZIF-8, ZIF-8/PEG, and Hbx@ZIF-8/PEG NPs (x = 0.6, 3, 6, 13, 25) (20 μL, 1 mg mL^−1^ in MQ) were added in triplicate to a transparent 96-well plate (Corning™ Costar™, Cell Culture Treated, Flat-Bottom) along with increasing concentrations of *p*-NPA (20 μL, 0−100 mM in EtOH). The volume was adjusted to 200 μL with MQ. As positive control, CA (20 μL, 50 μg mL^−1^ in TRIS) was also exposed to increasing concentrations of *p*-NPA (20 μL, 0–100 mM in EtOH) but the volume was adjusted to 200 μL using TRIS buffer (25 mM, pH 7.4) instead of MQ. Background correction was performed by replacing *p*-NPA with an equal volume of EtOH. The Abs at 400 nm was monitored over 90 min at RT. The concentration of *p*-NP was calculated using the molar extinction coefficient (*ε*) of *p*-NP (*ε* = 12 700 M^−1^ cm^−1^) and pathlength correction [21]. The reaction rates (V) for each *p*-NPA concentration were determined and fitted to a Michaelis-Menten kinetic model using a nonlinear curve-fitting algorithm in OriginPro 2024 software. The Michaelis-Menten parameters, i.e. maximum velocity (V_max_) and Michaelis-Menten constant (K_m_) were extracted from the fitted formula and the catalytic constant (K_cat_) and efficiency constant (K_eff_) were obtained using the following formulas.

(6) Michaelis-Menten formula: V = V_max_ × [*p*-NPA]/(K_m_ + [*p*-NPA])

(7) K_cat_ = V_max_/[E]; being [E] the molar concentration of the enzyme or the Zn molar concentration in the NPs.

(8) $$K_{\text{eff}}=K_{\text{cat}}/K_{m}$$

The fitting robustness was assessed using ANOVA F-tests (Table S1, Supporting Information).

#### 2.5.5 RBCs catalytic activity

The experiments were carried out with modifications to a reported protocol [27]. RBCs were isolated from bovine blood. Briefly, the blood was washed (3 × , 2000*g*, 20 min, 4 °C) with PBS (10 mM) and NaCl (0.9 %, 1:4 v/v) using a high-speed centrifuge (SL16R centrifuge, Thermo Scientific, Hvidovre, DK). Plasma was removed and the RBCs-containing pellet was diluted threefold in PBS. RBCs concentration was determined by freeze-drying a known volume and measuring its mass.

Calcein-AM (50 μL, 100 μM in PBS) was added to RBC suspensions (0.05, 0.1, 0.5 and 1 mg mL^−1^ in PBS), PBS was added to a final volume of 1 mL and incubated (37 °C, 300 rpm, 1 h) using a thermoshaker (MultiTherm Shaker, Benchmark Scientific, Sayreville, US). Next, the suspensions were spun down (1 × , 13 400 rpm, 5 min, RT) and the supernatant was collected. The RBCs of the pellet were lysed by adding 1 mL of cold MQ and vortexing (Vortex-Genie 2, Scientific Industries, Inc., Bohemia, US). Cell debris was removed by centrifugation (5 min, 13 400 rpm, RT) and the supernatant was collected. The supernatants (200 μL) were transferred in triplicate to a black 96-well plate (Nunc™ F96 MicroWell™, ThermoFisher, Roskilde, DK) and the fluorescence intensity was recorded with λ_ex_/λ_em_ = 485/528 nm with 20 nm bandwidth and gain set to 49 for calcein-AM measurements using a plate reader (Tecan Spark, Tecan Group Ltd., Männedorf, CH).

Hb6@ZIF-8/PEG NPs (0.05, 0.1, 0.5 and 1 mg mL^−1^ in MQ) and Hb (0.05, 0.1, 0.5 and 1 mg mL^−1^ in PBS) were incubated with calcein-AM (50 μL, 100 μM in MQ) in a total volume of 1 mL (1 h, 37 °C, 300 rpm) in a thermoshaker (MultiTherm Shaker, Benchmark Scientific, Sayreville, US). CA (0.05, 0.1, 0.5 and 1 mg mL^−1^ in PBS) was incubated with calcein-AM (50 μL, 100 μM in MQ) in a total volume of 1 mL (1 h, 37 °C, 300 rpm) in a thermoshaker (MultiTherm Shaker, Benchmark Scientific, Sayreville, US). Next, Hb6@ZIF-8/PEG NPs were separated by centrifugation (1 × , 13 400 rpm, 5 min, RT) and the supernatant was collected to measure its fluorescence. Hb and CA were measured directly. Blanks were prepared by adding MQ instead of calcein-AM and controls using PBS or MQ with calcein-AM only were included.

### 2.6 Oxygen transport ability of the Hbx@ZIF-8/PEG NPs (x = 0.6, 3, 6, 13, 25)

Oxygen transport ability of the Hbx@ZIF-8/PEG NPs was determined by UV–vis spectroscopy with modifications to a previous study [28]. Hb (3 mL, 0.05–0.7 mg mL^−1^ in PBS or MQ) and Hbx@ZIF-8/PEG NPs (3 mL, 0.02–0.1 mg mL^−1^ in MQ) spectra were recorded using a UV–vis spectrophotometer (UV-2600, Shimadzu Corp., Kyoto, JP) and quartz cuvettes (High Performance Quartz Glass, 10 × 10 mm, 3500 μL, Hellma GmbH & Co. KG, Müllheim, DE). Next, the Hb solution and Hbx@ZIF-8/PEG NPs were bubbled with nitrogen gas (N_2_) for 5 min, followed by the addition of a pinch of SDT to remove any residual oxygen and the spectrum of deoxygenated-Hb (deoxy-Hb) was recorded. Subsequently, the same Hb solution and the Hbx@ZIF-8/PEG NPs were bubbled for 5 min with air and the spectrum of oxygenated Hb (oxy-Hb) was recorded. As a control, ZIF-8/PEG NPs absorption spectrum was also recorded.

## 3 Results and discussion

### 3.1 Physicochemical characterization of ZIF-8 and ZIF-8/PEG NPs

Recently, our group reported a simple and efficient one-pot method for encapsulating Hb within ZIF-8/PEG NPs that exhibited an impressive 95 % oxyHb content, an encapsulation efficiency of 85 %, and notable resistance to Hb oxidation into methemoglobin (metHb). Notably, the addition of PEG played a crucial role in enhancing Hb entrapment within ZIF-8 [16]. PEG, a well-established FDA-approved biopolymer with extensive use in drug delivery, enhances the local Zn^2+^ concentration through weak coordination with its ethoxy groups [16,29]. Thanks to this affinity for Zn^2+^, PEG promotes the formation of pre-nucleation clusters, thereby facilitating the assembly of ZIF-8. Importantly, PEG interacts with ZIF-8 nuclei, guiding crystal growth along specific planes. Since the crystalline nature of MOFs is one of their most remarkable attributes, this enhanced crystallization has significant implications for various applications, including CO_2_ capture, catalysis, and sensing [30]. Additionally, the presence of PEG enables the synthesis of ZIF-8 NPs in water at room temperature, representing a critical advantage for the in-situ encapsulation of fragile biomolecules, such as Hb.

**Fig. 1:**
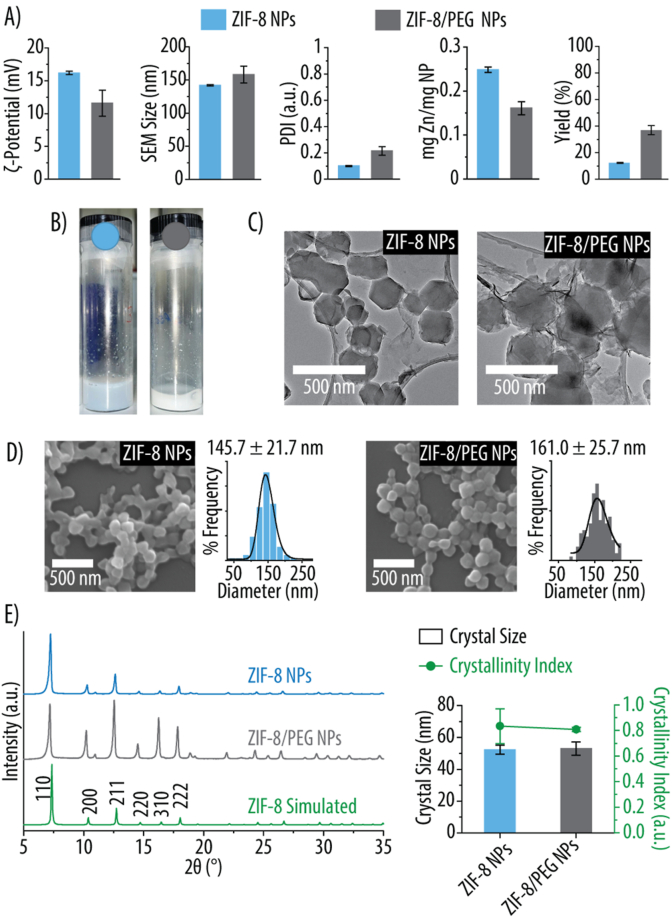
Characterization of ZIF-8 and ZIF-8/PEG NPs. **A)** Zeta-(ζ)-potential in mV, polydispersity index (PDI), zinc (Zn) content determined by inductively coupled plasma mass spectrometry, and synthesis yield of ZIF-8 and ZIF-8/PEG NPs. **B)** Photographic images of ZIF-8 and ZIF-8/PEG NPs suspensions in Milli-Q water (MQ). **C)** Transmission electron microscopy images of ZIF-8 and ZIF-8/PEG NPs. **D)** Scanning electron microscopy images with corresponding size distribution histograms, including mean particle size for ZIF-8 and ZIF-8/PEG NPs. **E)** X-ray diffraction spectra, crystallinity index, and crystallite size derived from the spectra for ZIF-8 and ZIF-8/PEG NPs. The simulated sodalite ZIF-8 pattern is included as a reference.

Fig. 1 presents the physicochemical characterization of ZIF-8 and ZIF-8/PEG NPs synthesized in MetOH and aqueous conditions, respectively. Both NPs display a positive ζ-potential, though the inclusion of PEG reduces it by 38 % (Fig. 1A), likely due to the presence of unprotonated hydroxyl groups in PEG, which lower the overall surface charge. The PDI for ZIF-8 NPs is 0.10, indicating a monodisperse distribution. In contrast, ZIF-8/PEG NPs exhibit a higher PDI of 0.22. Nonetheless, this value remains very close to 0.2, which is set as the threshold for pharmaceutic applications [31]. For ZIF-8/PEG NPs, the Zn content decreases by 35 %, probably due to PEG incorporation into the NP structure. However, despite using more reagents and a shorter reaction time, the overall formation yield increases in the presence of PEG, indicating improved reaction efficiency. The NPs dispersion in MQ is shown in Fig. 1B. TEM micrographs reveal that both ZIF-8 and ZIF-8/PEG NPs maintain a polyhedral/angular morphology (Fig. 1C). SEM images and size distribution analysis confirm that both NP types exhibit similar morphologies with an average size of ∼150 nm, an optimal range for intravenous administration (Fig. 1D) [32]. XRD spectra verify that both NPs retain the characteristic sodalite (SOD) crystal structure of ZIF-8 (Fig. 1E). For reference, the simulated XRD pattern of the SOD crystal structure (I $\bar{43}$ m crystal system, unit cell dimension *a* = 17.0095 Å at 298 K) is included [25]. The calculated crystal sizes for ZIF-8 and ZIF-8/PEG NPs are 52.3 ± 2.9 nm and 53.0 ± 4.2 nm, respectively, with both displaying a crystallinity index above 0.8 (Fig. 1E). Considering that the overall NP sizes are approximately 150 nm, these results suggest that the NPs are polycrystalline. The presence of multiple crystallites within a single NP can lead to structural defects, which may enhance catalytic activity by providing additional active sites [33,34].

These findings demonstrate that PEG incorporation does not significantly alter the structural properties of ZIF-8 NPs. However, it significantly enhances reaction efficiency by reducing synthesis time and precursor consumption. Most importantly, the ability to synthesize ZIF-8 NPs under aqueous conditions, enabled by PEG, paves the way for the in-situ encapsulation of fragile (bio)molecules, broadening their potential for biomedical applications.

### 3.2 Carbonic anhydrase-like activity of ZIF-8 and ZIF-8/PEG NPs

Nanozymes, nanomaterials with enzyme-like activities, have garnered significant attention for their diverse applications in molecular detection [35], bactericidal activity [36] and cancer therapy among others [15,21,37]. Unlike biological enzymes, which are expensive, challenging to produce, and prone to instability, nanozymes offer several advantages: they exhibit strong resistance to biological degradation, maintain functionality even after freeze-drying, and allow for structural customization through cost-effective synthesis [15,38]. Moreover, nanozymes demonstrate superior recyclability compared to enzymes, a crucial factor for prolonged catalytic applications. Thus, extensive efforts have been dedicated to designing and fabricating nanozyme candidates exploring various materials such as metal oxides, noble metals, and MOFs [39,40]. In particular, MOFs, composed of metal ions coordinated to organic ligands, provide high surface areas and serve as promising platforms for metalloenzyme mimetics [21,41].

Recently, ZIF-8 NPs have been reported to mimic CA activity due to their structural resemblance to the enzyme's active center, namely a Zn^2+^ metallic center coordinated to imidazolate. Native CA is a metalloenzyme that catalyzes the reversible hydration of CO_2_ to HCO_3_^−^. Its active site, Zn(Histidine (His))_3_OH_2_, consists of a tetrahedrally coordinated Zn^2+^ ion with three histidine imidazoles and one H_2_O molecule [42]. Coordination to the Lewis acid Zn^2+^ center lowers the pK_a_ of H_2_O from 14 to 6.8, facilitating the formation of Zn(His)_3_OH^−^, where the coordinated hydroxide ion can perform a nucleophilic attack on CO_2_ to generate HCO_3_^−^. The similarity between CA and ZIF-8 arises from their coordination environments: ZIF-8 NPs feature Zn^2+^ ions tetrahedrally coordinated by up to four HmIm ligands, forming a microporous SOD topology [43]. Some surface Zn^2+^ ions that are coordinated by fewer ligands could further contribute to the catalytic activity [21].

**Fig. 2:**
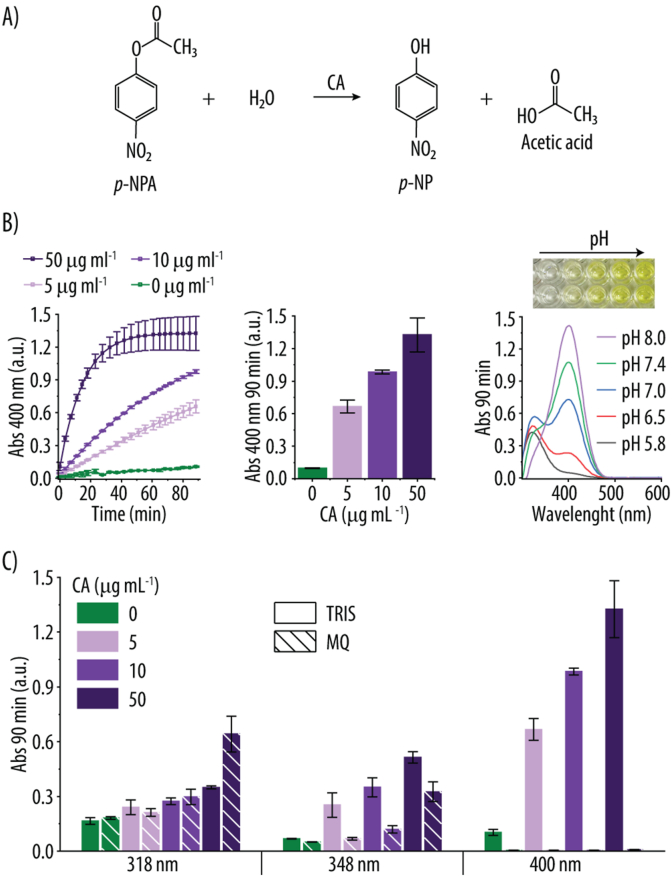
**A)** Schematic illustration of the hydrolysis of *p*-nitrophenyl acetate (*p*-NPA) into *p*-nitrophenol (*p*-NP) and acetic acid catalyzed by carbonic anhydrase (CA). **B)** Absorbance (Abs) readings over time at 400 nm following incubation of various CA concentrations with *p*-NPA (0.2 mM) in TRIS buffer (20 mM, pH 7.4) and at room temperature (RT). Abs readings at 400 nm after 90 min of incubation of different CA concentrations with *p*-NPA (20 mM) in TRIS buffer (20 mM, pH 7.4) at RT. UV–vis spectra after 90 min incubation of CA (50 μg mL^−1^) with *p*-NPA (0.2 mM) at different pH values (5.8–8.0) and at RT. Inset: Photograph of the reaction wells showing increasing yellow coloration with rising pH. **C)** Abs signals at 318, 348 and 400 nm after 90 min of reaction of different CA concentrations with *p*-NPA (0.2 mM) in 20 mM TRIS buffer at pH 7.4 (solid bars) and in MilliQ (MQ) water (stripped bars) at RT.

To evaluate the CA-like activity of ZIF-8 and ZIF-8/PEG NPs, we used *p*-NPA as a colorimetric substrate, given its hydrolysis mechanism, which parallels CO_2_ hydration but also enables convenient and accurate measurements (Fig. 2A) [44]. As expected, incubation of CA with *p*-NPA (0.2 mM) resulted in an increase in Abs at 400 nm, indicating the formation of the yellow *p*-nitrophenol (*p*-NP) product (Fig. 2B). Moreover, higher CA concentrations accelerated *p*-NP formation (Fig. 2B). Since *p*-NP absorbance is pH-dependent, maximizing at 318 nm under acidic conditions and shifting to 400 nm at neutral to basic pH (≥7.4), with an isosbestic point at 348 nm (Fig. 2B) [45], we measured the Abs signal at all three wavelengths in two solvents: TRIS (20 mM, pH 7.4) and MQ (Fig. 2C). The highest Abs signal was observed at 400 nm, consistent with *p*-NP's stronger absorption in neutral/basic environments (*ε* = 12 700 cm^−1^ M^−1^) [21]. Furthermore, measuring CA activity at pH 7.4 is preferable, as it better reflects physiological conditions for enzyme activity studies.

**Fig. 3:**
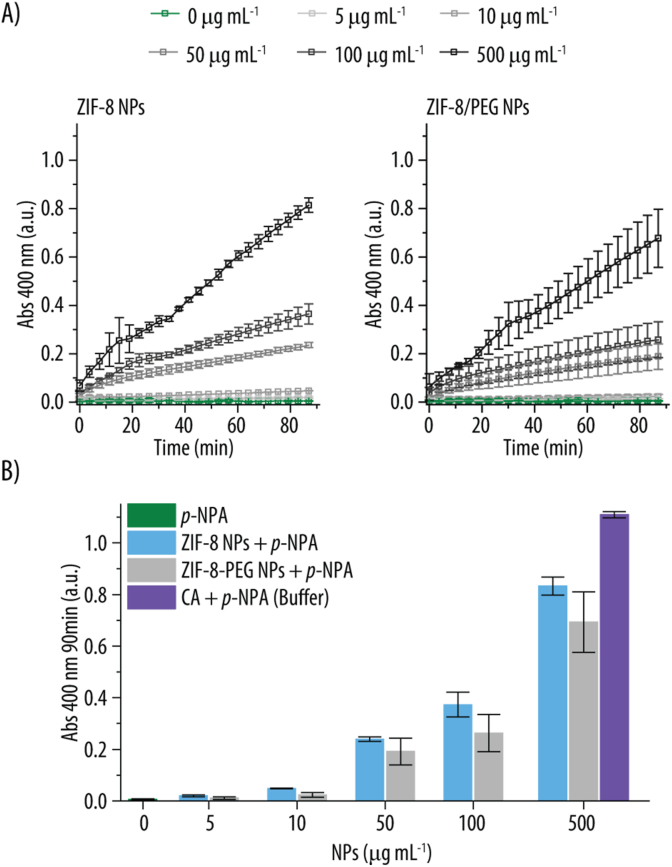
**A)** Absorbance (Abs) readings over time at 400 nm following incubation of *p*-nitrophenyl acetate (*p*-NPA) (0.2 mM) with various concentrations of ZIF-8 NPs and ZIF-8/PEG NPs. The reaction was conducted in Milli-Q (MQ) water at room temperature (RT). **B)** Abs readings at 400 nm after 90 min of incubation of different concentrations of ZIF-8 and ZIF-8/PEG NPs with *p*-NPA (0.2 mM) in MQ water at RT. As controls, *p*-NPA only in MQ water and carbonic anhydrase (CA) incubated with *p*-NPA (0.2 mM) in TRIS buffer (20 mM, pH 7.4) at RT are shown.

Next, we evaluated the ability of ZIF-8 and ZIF-8/PEG NPs to facilitate the hydrolysis of *p*-NPA, similar to this function of CA. For that, we incubated increasing concentrations of ZIF-8 and ZIF-8/PEG NPs to 0.2 mM *p*-NPA at RT and measured the Abs signal at 400 nm over time. Due to the lack of stability of ZIF-8 and ZIF-8/PEG NPs in buffers that mimic physiological conditions, the reaction was conducted in MQ water. Fig. 3A demonstrates that both ZIF-8 and ZIF-8/PEG NPs effectively catalyze the conversion of *p*-NPA into *p*-NP, with increasing NP concentration enhancing the catalytic efficiency. This observation aligns with findings from previous studies in which ZIF-8 catalyzed the *p*-NPA hydrolysis [21,46]. Fig. 3B shows a comparison of the catalytic activity of ZIF-8, ZIF-8/PEG NPs and CA at different concentrations and after 90 min of reaction. Since CA displays the highest activity in conditions similar to physiological ones, the CA activity was measured at pH 7.4 (TRIS buffer). The results show how, for all the studied concentrations, ZIF-8 NPs displayed slightly higher activity than ZIF-8/PEG NPs. For example, at 100 μg mL^−1^ of NPs, the Abs at 400 nm at 90 min, is a 30 % lower for the ZIF-8/PEG NPs. We attribute this lower catalytic activity for ZIF-8/PEG NPs to the lower Zn content. As shown in Fig. 1A, for 1 mg of NPs, the Zn content for ZIF-8 NPs is of 0.25 mg, while for ZIF-8/PEG NPs the content is only of 0.16 mg Zn. This is an important fact since the Zn^2+^ coordinated to His (i.e., Zn(His)_3_OH^−^) is believed to be the active catalytic site. Thus, the higher Zn content in ZIF-8 NPs could contribute to their enhanced catalytic performance [46]. These findings suggest that the structural composition and metal content of the NPs play a crucial role in their enzymatic mimicry. The catalytic activity of the natural CA enzyme is markedly higher than that of ZIF-8 and ZIF-8/PEG NPs. Thus, at a concentration of 50 μg mL^−1^, the *p*-NP absorption at 400 nm is 82 % and 85 % higher for the natural CA enzyme compared to ZIF-8 and ZIF-8/PEG NPs, respectively. However, the catalytic activity of both ZIF-8 and ZIF-8/PEG NPs approaches that of the natural CA enzyme when increasing concentration. For instance, at a concentration of 500 μg mL^−1^, the enzymatic activity is only 25 % and 37 % lower, for ZIF-8 and ZIF-8/PEG NPs, respectively, than that of the CA enzyme (Fig. 3B).

### 3.3 Physicochemical Characterization of Hbx@ZIF-8/PEG NPs (x = 0.6, 3, 6, 13, 25)

**Fig. 4:**
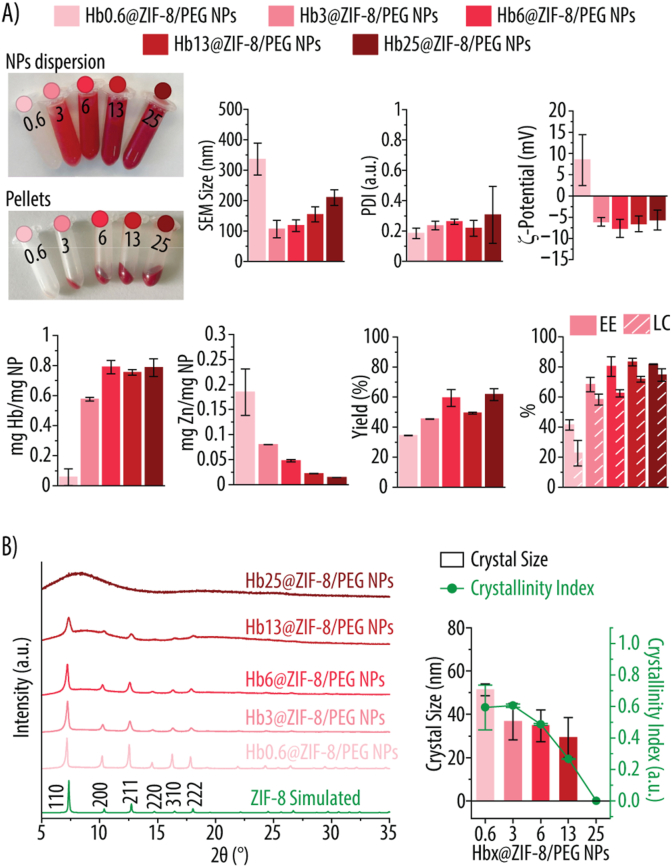
Characterization of Hbx@ZIF-8/PEG NPs (x = 0.6, 3, 6, 13, 25). **A)** Photographic images of the NPs suspensions in Milli-Q water (MQ) and the corresponding pellets after centrifugation, NPs size (nm) measured by scanning electron microscopy (SEM), polydispersity index (PDI), zeta-(ζ)-potential in mV, Hb and zinc (Zn) content determined by inductively coupled plasma mass spectrometry, synthesis yield, encapsulation efficiency (EE) and loading content (LC) of Hbx@ZIF-8/PEG NPs. **B)** X-ray diffraction (XRD) spectra crystallinity index and crystallite size obtained from the XRD spectra. x = 0.6, 3, 6, 13, 25 indicates the concentration of Hb in mg mL^−1^ used for the NPs assembly.

Once the CA-like activity of ZIF-8 and ZIF-8/PEG NPs had been identified, we incorporated increasing amounts of Hb to create a novel HBOCs with the ability to transport both CO_2_ and oxygen. Since we have previously demonstrated that the presence of PEG is essential to encapsulate large amounts of Hb, only ZIF-8/PEG NPs were considered [16]. For that, increasing concentrations of Hb were added to the Zn^2+^, HmIm and PEG mixture to render Hbx@ZIF-8/PEG NPs, where x indicates the concentration of Hb used for their assembly (x = 0.6, 3, 6, 13, 25 mg mL^−1^). Fig. 4 shows images of the different Hbx@ZIF-8/PEG NPs suspensions and the corresponding pellets, along with their physicochemical characterization. The dispersion and pellet of Hb0.6@ZIF-8/PEG NPs display a faint pink color, indicating a low Hb content. As the Hb concentration used for their assembly increases, the red color of both dispersion and pellet intensifies, suggesting a higher degree of Hb loading. Additionally, pellet size visibly increases with higher initial Hb concentrations (Fig. 4A). SEM size analysis shows how Hb0.6@ZIF-8/PEG form the largest NPs with a diameter of ∼340 nm (Fig. 4A). However, increasing the Hb amount from 0.6 to 3 mg mL^−1^ results in a size decrease to 105 ± 29 nm. Further increasing the Hb concentration to 6, 13 and 25 mg mL^−1^ resulted on a slight size increase to 117, 154 and 210 nm, respectively. Sizes from 150 to 200 nm are considered ideal for HBOC development. Smaller NPs (<100 nm) can penetrate in between the gaps of endothelial cells, extravasating and scavenging the nitric oxide (NO) in the underlaying smooth muscle. Being NO a vasodilator, its extravasation results in vasoconstriction and hypertension [3]. In contrast, larger particles (1–3 μm) undergo phagocytosis [47]. The PDI of the different Hbx@ZIF-8/PEG NPs obtained remains around 0.2 indicating an acceptable size distribution for pharmaceutical use (Fig. 4A). In contrast to ZIF-8/PEG NPs, which displayed a positive ζ-potential (of ∼12 mV, Fig. 1A), Hbx@ZIF-8/PEG NPs display a negative ζ-potential of around −7 mV, which can be attributed to Hb's negative surface charge in MQ (Fig. 4A). An exception is Hb0.6@ZIF-8/PEG NPs, which display a positive ζ-potential of 8.5 mV, which can be attributed to the low Hb concentration used for their preparation, which is not sufficient to counteract the positive charge of the ZIF-8/PEG NPs [48]. ICP-MS was next used to assess the amount of Zn and Fe within the different Hbx@ZIF-8/PEG NPs, which was subsequently used to calculate their yield, EE and LC. As the Hb concentration increases, the Zn content in the NPs decreases, which is expected as more Hb incorporates into the NPs increasing their overall mass (Fig. 4A). Interestingly, as the amount of Zn per NP increases, the yield of Hbx@ZIF-8/PEG NPs formation also increases, suggesting that Hb may facilitate interactions between Zn^2+^ and HmIm, promoting the ZIF-8 assembly (Fig. 4A). ICP-MS Fe content analysis reveals that higher Hb concentrations used for the assembly lead to greater Hb incorporation into the NPs, with a maximum loading of 0.79 ± 0.04 mg Hb/mg NP obtained for Hb25@ZIF-8/PEG NPs (Fig. 4A) [49]. The EE and LC also increased the higher the Hb concentration used for the Hbx@ZIF-8/PEG NPs assembly (Fig. 4A). EE and LC as high as 83 and 75 %, respectively, were obtained. While achieving EE is important due to economic implications, high LC is essential for achieving comparable oxygen carrying capacities to natural blood, since Hb constitutes about 96 % of the dry weight in native RBCs [50,51]. PXRD spectra show how increasing the Hb concentration results in a loss of crystallinity (Fig. 4B). Thus, the diffraction peaks become less and less prominent as the concentration of Hb increases suggesting that the incorporation of Hb may disrupt the crystalline arrangement of Zn^2+^ and HmIm. For Hb13@ZIF-8/PEG NPs a diffractogram's hump indicating an amorphous phase can be detected and this hump becomes completely dominant for Hb25@ZIF-8/PEG NPs, where no reflection peaks can be detected. The crystallinity index and crystal size decrease as the Hb content increases, likely due to the interference of Hb molecules in the nucleation and growth of ZIF-8 (Fig. 4B). A decrease in the crystal size indicates that Hb can act as a nucleation site, promoting the formation of more but smaller crystals while simultaneously hindering their growth by blocking active sites. Additionally, Hb molecules incorporate into the growing framework, disrupting the periodic arrangement of Zn^2+^ and HmIm, leading to structural distortion and a lower degree of crystallinity [52,53].

**Fig. 5:**
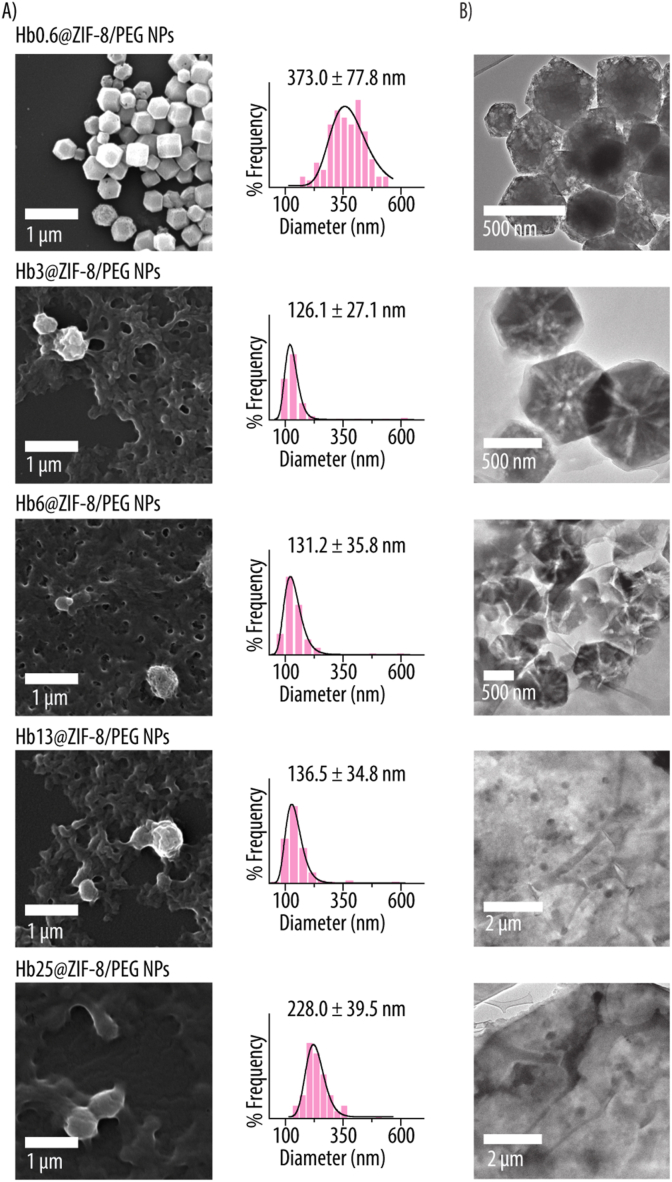
Characterization by electron microscopy of Hbx@ZIF-8/PEG NPs (x = 0.6, 3, 6, 13, 25). **A)** Scanning electron microscopy micrographs with corresponding size distribution histograms, including mean particle size. **B)** Transmission electron microscopy images. x = 0.6, 3, 6, 13, 25 indicates the concentration of Hb in mg mL^−1^ used for the NPs assembly.

The morphology and size of Hbx@ZIF-8/PEG NPs were analyzed using SEM and TEM (Fig. 5). SEM micrographs show how Hb0.6@ZIF-8/PEG NPs exhibit a polyhedral morphology, indicating that for the lowest Hb concentration, the characteristic ZIF-8 NPs morphology is preserved (Fig. 5A). However, as the Hb content increases, the Hbx@ZIF-8/PEG NPs adopt a more spherical shape. The size histograms obtained from SEM imaging indicate a sharp size decrease (i.e., from 373 to 126 nm) for a Hb increase from 0.6 to 3 mg mL^−1^. Further increase in the Hb concentration results in a size increase with Hb6@ZIF-8/PEG, Hb13@ZIF-8/PEG and Hb25@ZIF-8/PEG NPs displaying mean average sizes of 131, 136 and 228 nm, respectively. TEM images confirm these observations, showing that the polyhedral shape of ZIF-8 progressively transitions to a spherical morphology as the Hb content increases (Fig. 5B). These findings are likely due to Hb interfering with crystal growth, leading to structural rearrangement and size reduction [52].

These results confirm that Hbx@ZIF-8/PEG NPs exhibit tunable size, morphology, and high Hb loading efficiency, features highly desirable for HBOC development. As with other ZIF-8 systems, potential immune interactions and Zn^2+^ release are important considerations [54,55]. To address the former, we developed dual metal phenolic networks (MPN) and PEG coatings, which stabilize Hbx@ZIF-8/PEG NPs in physiological media, enhance biocompatibility and protect encapsulated Hb [17,18]. In particular, these coatings reduced Hb release and minimized interference with the coagulation cascade, as demonstrated by stable prothrombin and activated partial thromboplastin times. Complement activation assays revealed only slight increases in C5a levels, suggesting a low risk of severe immune reactions. *In vivo*, MPN-coated and PEGylated Hb-loaded ZIF-8 NPs displayed extended circulation times, with significantly longer half-lives than free Hb. The effect of the MPN and PEG coatings on the Zn^2+^ leaching remains to be fully clarified; however, if this proves problematic, mitigation strategies such as substituting Zn^2+^ with the more biocompatible Mg^2+^ could be pursued.

### 3.4 Carbonic anhydrase-like activity of Hbx@ZIF-8/PEG NPs (x = 0.6, 3, 6, 13, 25)

**Fig. 6:**
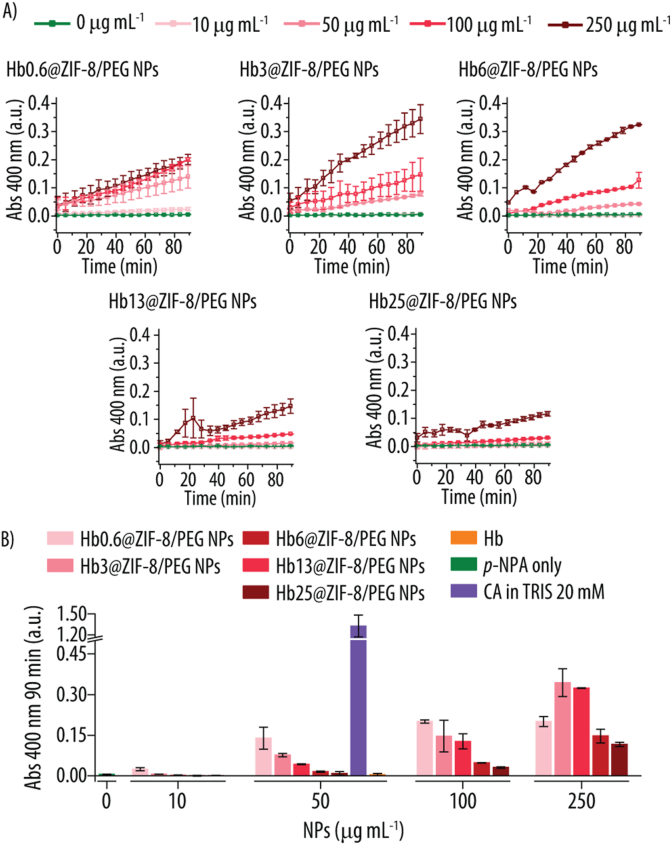
**A)** Absorbance (Abs) readings over time at 400 nm following incubation of *p*-nitrophenyl acetate (*p*-NPA) (0.2 mM) with various concentrations of Hb0.6@ZIF-8/PEG, Hb3@ZIF-8/PEG, Hb6@ZIF-8/PEG, Hb13@ZIF-8/PEG and Hb25@ZIF-8/PEG NPs. The reaction was conducted in Milli-Q (MQ) water at room temperature (RT). **B)** Abs readings at 400 nm after 90 min of incubation of different concentrations of Hbx@ZIF-8/PEG NPs with *p*-NPA (0.2 mM) in MQ water at RT. As controls, *p*-NPA only in MQ water and carbonic anhydrase (CA) incubated with *p*-NPA (0.2 mM) in TRIS buffer (20 mM, pH 7.4) at RT are shown. x = 0.6, 3, 6, 13, 25 indicates the concentration of Hb in mg mL^−1^ used for the NPs assembly.

We next assessed whether the incorporation of Hb had any effect on the CA-like activity of ZIF-8/PEG NPs. For that, we also monitored the Abs signal at 400 nm over time upon incubating the different Hbx@ZIF-8/PEG NPs with *p*-NPA for up to 90 min. Fig. 6A shows an increase in Abs signal over time for all the Hbx@ZIF-8/PEG NPs, and this increase in *p*-NP formation is higher as the NPs concentration increases. Thus, this result demonstrated that Hbx@ZIF-8/PEG NPs also display CA-mimetic properties. We note that, due to the limited stability of Hbx@ZIF-8/PEG NPs in buffers mimicking physiological conditions, the reaction was performed in MQ water [16]. Nonetheless, we have developed strategies (i.e., dual MPN and PEG coatings) that render Hb@ZIF-8/PEG NPs stable in commonly used buffers, including PBS and cell culture media [17,18].

The CA-like activity of Hbx@ZIF-8/PEG NPs is further confirmed in Fig. 6B, where the Abs 400 nm at 90 min of reaction is plotted. Interestingly, the higher the Hb content in the NPs, the slower the reaction. Thus, at 100 μg mL^−1^, Hb0.6@ZIF-8/PEG NPs show an Abs signal of 0.2, while the Abs signal for Hb25@ZIF-8/PEG NPs, the highest studied Hb concentration is only of 0.1. Since free Hb does not exhibit CA-like activity (see Fig. S2, Supporting Information), this suggests that the Zn quantity is directly related to the reaction rate, as Zn is believed to be the active site for the reaction. Furthermore, ICP-MS analysis of the Hb stock revealed Zn levels comparable to those of the blanks, in agreement with the absence of significant CA in the Hb stock.

### 3.5 Enzyme kinetics and oxygen transport ability of ZIF-8, ZIF-8/PEG and Hbx@ZIF-8/PEG NPs (x = 0.6, 3, 6, 13, 25)

**Fig. 7:**
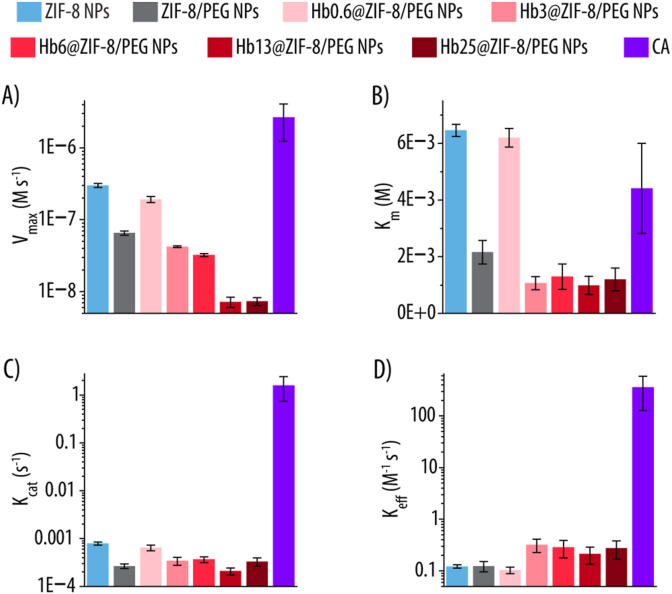
Michaelis-Menten parameters of ZIF-8, ZIF-8/PEG, and Hbx@ZIF-8/PEG NPs (x = 0.6, 3, 6, 13, 25) at 100 μg mL^−1^ incubated with *p*-nitrophenyl acetate (*p*-NPA) all in Milli-Q water and room temperature (RT). As a positive control, carbonic anhydrase (CA) at 50 μg mL^−1^ with *p*-NPA all in TRIS buffer (20 mM, pH 7.4) and RT was considered. x = 0.6, 3, 6, 13, 25 indicates the concentration of Hb in mg mL^−1^ used for the NPs assembly. **A)** Maximum velocity (V_max_), **B)** Michaelis-Menten constant (K_m_), **C)** catalytic constant (turnover number) (K_cat_) and **D)** efficiency constant (K_eff_ = K_cat_/K_m_) are shown.

Upon confirming that not only ZIF-8 and ZIF-8/PEG NPs, but also the Hb-loaded Hbx@ZIF-8/PEG NPs were able to hydrolyze *p*-NPA into *p*-NP, our next objective was to assess whether they also displayed an enzyme-like behavior. For that, we evaluated whether the reaction kinetics of the different NPs could be fitted into a Michaelis-Menten model, which describes the kinetics of enzyme-catalyzed reactions, where an enzyme (or catalyst) binds to a substrate to form an enzyme-substrate (ES) complex, which then converts into a product (P) (Fig. S3A). For that, we exposed 100 μg mL^−1^ of the different NPs (i.e., ZIF-8, ZIF-8/PEG and Hbx@ZIF-8/PEG NPs) to varying concentrations of *p*-NPA (0–10 mM) and we plotted the reaction velocity (V) against the initial concentration of *p*-NPA and fitted the curves to the Michaelis-Menten equation. As a positive control, we also performed the experiment using natural CA at a concentration of 50 μg mL^−1^. Fig. S3B shows how, for all the different NPs, the V increases with the substrate concentration (i.e., *p*-NPA), indicating that the active sites are fully occupied. This, together with the hyperbolic curve, suggests Michaelis-Menten behavior. The coefficients of determination (R^2^) are above 0.99 for both ZIF-8, ZIF-8/PEG and Hb6@ZIF-8/PEG NPs indicating an excellent fit, suggesting strong adherence to Michaelis-Menten kinetics. Slightly lower, but still good fit R^2^ (still above 0.94 in all cases) are observed for Hb0.6@ZIF-8/PEG, Hb3@ZIF-8/PEG, Hb13@ZIF-8/PEG and Hb25@ZIF-8/PEG NPs. Table S1 (Supporting Information) reports ANOVA F-tests for all Michaelis-Menten fittings, with all p(F) values < 0.05, demonstrating the robustness of the kinetic analysis. Fig. 7 and Table 1 show the different Michaelis-Menten parameters extracted from the fitting compared to those of natural CA. The maximum velocity (V_max_), which indicates the maximum rate of reaction where all catalytic sites are saturated with substrate (i.e., when all the enzymes are in the ES complex), decreases with the addition of PEG (Fig. 7A). This could be attributed to the decreased Zn^2+^ content for the ZIF-8/PEG NPs as compared to ZIF-8 NPs, directly affecting the number of active sites. A similar trend was observed for Hb-containing NPs (i.e., Hbx@ZIF-8/PEG NPs), where a lower Zn^2+^ due to the increased amount of Hb resulted also in lower V_max_. As such, the higher the Hb concentration used to prepare the NPs, the lower the V_max_. The Michaelis-Menten constant (K_m_) (Fig. 7B), which is inversely proportional to the catalyst affinity for the substrate, indicated that the addition of PEG and increasing amounts of Hb led to an increase in substrate affinity (lower K_m_), possibly due to interactions between PEG and/or Hb and *p*-NPA. The catalytic constant or turnover number (K_cat_), which describes the number of substrate molecules converted per catalyst per unit of time, is calculated by dividing the V_max_ by the enzyme's molar concentration, which usually corresponds to the concentration of active sites. For most enzymes, including CA, the number of enzyme molecules equals the number of active sites, making it straightforward to obtain the K_cat_. In the case of nanozymes, calculating the number of active sites is more challenging, requiring certain assumptions [56]. Since ZIF-8 is a porous material, we assume that *p*-NPA molecules can access all the Zn atoms within the scaffold. Therefore, the concentration of active sites corresponds to the concentration of Zn atoms. K_cat_ (Fig. 7C) was calculated by dividing V_max_ by the Zn molar concentration determined by ICP-MS. Thus, K_cat_ showed a similar trend to V_max_. In either case, the presence of PEG or Hb may hinder *p*-NPA's access to the active sites. Finally, the catalytic efficiency constant (K_eff_), defined as K_cat_/K_m_ (Fig. 7D), reflects how effectively the catalyst converts the substrate into a product. NPs with higher Hb content exhibited greater catalytic efficiency, likely due to increased substrate affinity outweighing changes in K_cat_. Additionally, NPs possessing higher amounts of Hb not only will display improved O_2_ transport capacity but also enhanced potential to catalyze the conversion of CO_2_ into HCO_3_^−^.

**Table 1:** Michaelis-Menten parameters of ZIF-8, ZIF-8/PEG, and Hbx@ZIF-8/PEG NPs (x = 0.6, 3, 6, 13, 25) at 100 μg mL^−1^, incubated with *p*-nitrophenyl acetate (*p*-NPA) in Milli-Q water at room temperature (RT). Carbonic anhydrase (CA, 50 μg mL^−1^) incubated with *p*-NPA in TRIS buffer (20 mM, pH 7.4) at RT was used as a positive control. Here, x = 0.6, 3, 6, 13, 25 corresponds to the hemoglobin concentration (mg mL^−1^) employed during NPs assembly. Abbreviations: maximum velocity (V_max_), Michaelis–Menten constant (K_m_), catalytic constant (turnover number, K_cat_), and catalytic efficiency (K_eff_ = K_cat_/K_m_).

Enzyme/NPs	V_max_ (10^−8^ M s^−1^)	K_m_ (10^−3^ M^−1^)	K_cat_ (10^−4^ s^−1^)	K_eff_ (M^−1^ s^−1^)
ZIF-8 NPs	30.0 ± 2	6.5 ± 0.2	7.9 ± 0.6	0.12 ± 0.01
ZIF-8/PEG NPs	6.5 ± 0.4	2.2 ± 0.4	2.7 ± 0.3	0.12 ± 0.03
Hb0.6@ZIF-8/PEG NPs	19.2 ± 1.8	6.2 ± 0.3	6.4 ± 0.9	0.1 ± 0.01
Hb3@ZIF-8/PEG NPs	4.2 ± 0.1	1.1 ± 0.2	3.4 ± 0.6	0.32 ± 0.09
Hb6@ZIF-8/PEG NPs	3.2 ± 0.2	1.3 ± 0.4	3.7 ± 0.5	0.28 ± 0.11
Hb13@ZIF-8/PEG NPs	0.7 ± 0.1	1.0 ± 0.3	2.1 ± 0.4	0.21 ± 0.08
Hb25@ZIF-8/PEG NPs	0.7 ± 0.1	1.2 ± 0.4	3.3 ± 0.6	0.28 ± 0.11
CA	264.1 ± 141.2	4.4 ± 1.6	15821.7 ± 8461.9	358.36 ± 231.11

**Fig. 8:**
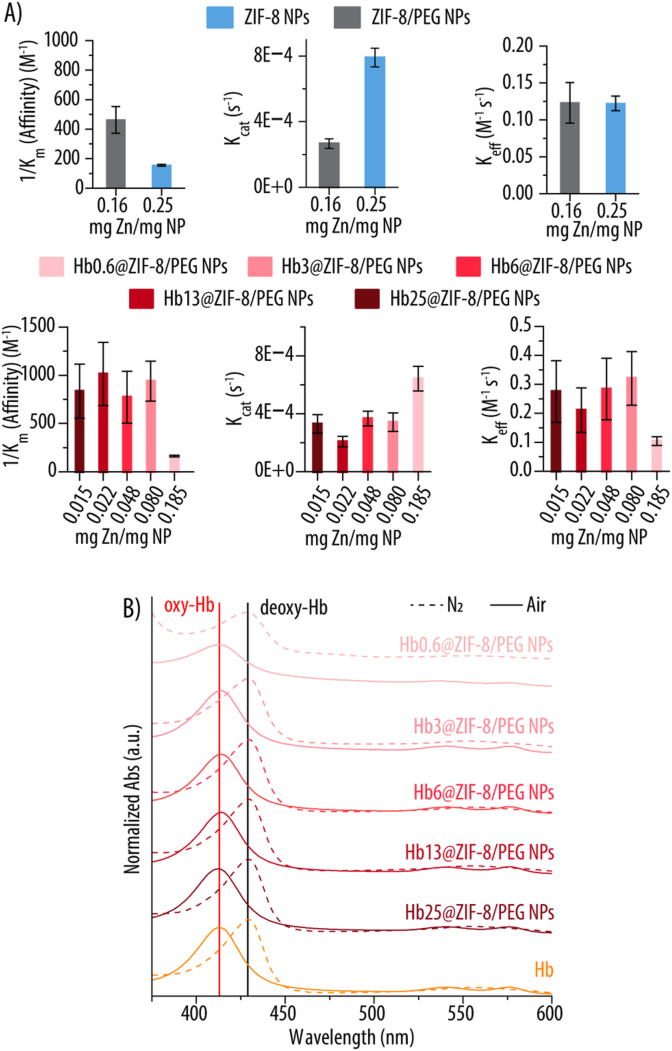
**A)** Michaelis-Menten kinetic parameters (i.e., inversed Michaelis-Menten constant (1 /K_m_) or affinity towards the substrate; catalytic constant (K_cat_) and efficiency constant (K_eff_)) as a function of zinc (Zn) content for ZIF-8 and ZIF-8/PEG NPs and Hb-loaded Hbx@ZIF-8/PEG NPs (x = 0.6, 3, 6, 13, 25). x = 0.6, 3, 6, 13, 25 indicates the concentration of Hb in mg mL^−1^ used for the NPs assembly. **B)** UV–Vis absorbance (Abs) spectra of Hbx@ZIF-8/PEG NPs and Hb in Milli-Q water after N_2_ purging (dashed line) in the presence of sodium dithionite as an oxygen scavenger and subsequent air purging (solid line). The absorbance maxima of deoxy-Hb (black) and oxy-Hb (red) are shown.

Fig. 8 shows how the Michaelis-Menten parameters vary with the Zn content in the NPs. The results confirm that incorporating PEG into ZIF-8 NPs enhances substrate affinity (Fig. 8A), likely due to PEG's higher affinity for *p*-NPA molecules [57]. However, as expected, the reduced Zn content leads to a lower catalytic constant (K_cat_), consistent with the role of Zn^2+^ ions as the active sites in the ZIF-8 structure [21]. As a result, the improved affinity observed in ZIF-8/PEG NPs and the higher turnover number for ZIF-8 NPs offset each other, yielding nearly identical K_eff_ for both NPs. A similar trend is observed for Hbx@ZIF-8/PEG NPs (Fig. 8A). Increasing the Hb content in the NPs enhances the affinity for *p*-NPA, which is expected, as proteins have been shown to improve the affinity of nanozymes towards the substrate [58]. This is likely due to the non-polar nature of *p*-NPA, which may interact with Hb's non-polar regions [59]. Interestingly, Hb0.6@ZIF-8/PEG NPs exhibit a K_cat_ over 40 % higher than that of the other Hb-containing NPs, likely due to their relatively higher Zn^2+^ content. In contrast, Hb3@ZIF-8/PEG, Hb6@ZIF-8/PEG, Hb13@ZIF-8/PEG, and Hb25@ZIF-8/PEG NPs show comparable K_cat_ values, suggesting a Zn^2+^ threshold, between 0.08 and 0.185 mg Zn per mg of NPs, below which the turnover number significantly declines. Despite the markedly higher K_cat_ of Hb0.6@ZIF-8/PEG NPs, its K_eff_ remains lower than that of the other Hbx@ZIF-8/PEG NPs, probably because, in the context of hydrolysis, increased substrate affinity becomes more critical than turnover rate. The ZIF-8, ZIF-8/PEG, and Hb0.6@ZIF-8/PEG NPs exhibited K_m_ comparable to the ones reported in the literature, indicating similar affinities toward *p*-NPA [17,43]. In contrast, the Hb3@ZIF-8/PEG, Hb6@ZIF-8/PEG, Hb13@ZIF-8/PEG, and Hb25@ZIF-8/PEG NPs demonstrated lower K_m_ values, suggesting enhanced substrate binding, probably due to the presence of Hb. However, the observed V_max_ were lower than those reported previously using the same NPs concentration (100 μg mL^−1^). Overall, these findings support the classification of ZIF-8 NPs, ZIF-8/PEG NPs, and Hbx@ZIF-8/PEG NPs as nanozymes, as they catalyze the same reaction as CA and follow a Michaelis-Menten model [40].

We next evaluated the oxygen transport capabilities of the different Hbx@ZIF-8/PEG NPs (Fig. 8B). To do this, we monitored spectral shifts in the Soret peak and Q-bands following one cycle of deoxygenation and oxygenation. The Abs spectra of ZIF-8/PEG, Hbx@ZIF-8/PEG NPs and untreated Hb are provided in Fig. S4 (Supporting Information). As shown, both free Hb and Hbx@ZIF-8/PEG NPs exhibit three characteristic peaks: a Soret peak at 413 nm and two Q-bands in the 530–580 nm range, indicating that the encapsulated Hb maintains its oxy-Hb form. It is worth noting that as the Hb loading decreases, the relative contribution of the ZIF-8/PEG matrix to the overall absorbance increases, making the Hb-specific peaks less distinguishable, particularly in the case of Hb0.6@ZIF-8/PEG NPs. Following N_2_ and air purging cycles, the Abs spectra of the NPs reveal more pronounced peaks (Fig. 8B), which we attribute to SDT-induced disassembly of the NPs (Fig. S4, Supporting Information). Despite this disassembly, the released Hb retains its functionality, as evidenced by a shift of the Soret peak to 429 nm and the merging of the two Q-bands into a single broad peak around ∼460 nm. Subsequent reoxygenation shifts the Soret band back toward 413 nm and restores the two distinct Q-bands, confirming that Hb's oxygen-binding ability remains intact.

**Fig. 9:**
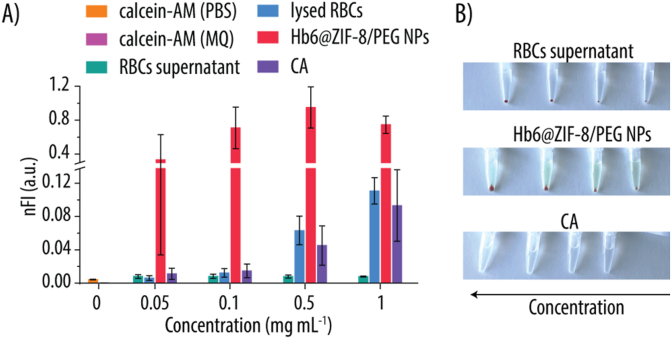
**A)** Normalized fluorescence intensity (nFI) readings of calcein-acetoxymethyl (calcein-AM) incubated with RBCs were measured in the supernatant (green) or in the cytosolic contents (blue) after lysing the cells. Calcein-AM incubated with Hb6@ZIF-8/PEG NPs (red), carbonic anhydrase (CA) (purple) and the controls of calcein-AM only in phosphate buffered saline (PBS) (orange) Milli-Q (MQ) (pink) are also shown. **B)** Photographs of the RBCs-, Hb6@ZIF-8/PEG NPs- and CA-containing Eppendorf after centrifugation.

Having confirmed that the Hbx@ZIF-8/PEG NPs support both CA-like activity and oxygen transport, we next investigated how their CA-like functionality compares to that of native RBCs (Fig. 9). We used calcein-AM, a cell-permeable, non-fluorescent dye that is converted into a fluorescent product by CA (Fig. S5, Supporting Information). For these assays, we selected Hb6@ZIF-8/PEG NPs formulation, which exhibited one of the highest K_cat_ among the tested Hbx@ZIF-8/PEG NPs (Fig. 7B–Table 1). K_eff_ was not considered here since the substrate was no longer *p*-NPA, and K_eff_ specifically reflects substrate affinity. Moreover, Hb6@ZIF-8/PEG NPs contain a high Hb loading and serve as a representative system for functional evaluation.

As shown in Fig. 9A, the Hb6@ZIF-8/PEG NPs produced an increasing normalized FI (**nFI**) signal with increasing concentrations, up to 0.5 mg mL^−1^, beyond which the signal plateaued. Control experiments confirmed that Hb alone does not produce any significant calcein AM fluorescence (Fig. S6, Supporting Information), indicating that the observed signal arises from the ZIF-8 component of the NPs. The RBC supernatant showed negligible FI signal, whereas lysed RBCs (containing cytosolic contents) yielded increasing FI signals with rising concentrations, consistent with intracellular CA-mediated conversion of calcein AM. CA alone also generated a concentration-dependent FI signal, though it was considerably lower than that produced by Hb6@ZIF-8/PEG NPs. This reduced efficiency is likely due to the larger size of calcein AM relative to *p*-NPA, which limits its accessibility to CA's relatively small active site, an adaptation optimized for the reversible hydration of CO_2_ (Fig. S5, Supporting Information).

Taken together, these results demonstrate that Hb6@ZIF-8/PEG NPs exhibit CA-like enzymatic activity and oxygen transport behavior comparable to that of native RBCs, underscoring their potential as effective synthetic RBC substitutes.

## 4 Conclusions

In this work, we demonstrated that ZIF-8/PEG NPs can act as dual-purpose Hb carriers, combining oxygen transport with CA-like activity. PEG incorporation improved synthesis efficiency and enabled high Hb loading with the resulting Hb@ZIF-8/PEG NPs retaining oxygen-binding capacity and enzymatic mimicry following Michaelis–Menten kinetics. These findings position ZIF-8–based systems as promising multifunctional HBOCs that address not only oxygen delivery but also CO_2_ transport, an essential yet often overlooked function of native RBCs.

Despite these advances, several challenges remain. The catalytic activity of Hb@ZIF-8/PEG NPs, although measurable, is still lower than that of native CA, and increasing Hb loading reduced Zn^2+^ availability and crystallinity, thereby compromising performance. Future work should focus on optimizing the balance between Hb encapsulation and catalytic efficiency. Moreover, because the present study was performed under *in vitro* conditions in MQ water, efforts should be directed toward enhancing nanoparticle stability in physiologically relevant media. Our previous work has shown that tailored surface coatings (e.g., dual MPN and PEG coatings) improve colloidal stability; here, systematic evaluation of how such modifications affect CA-like activity will be critical. Looking ahead, thorough biocompatibility studies in cell-based assays and animal systems are needed, particularly in disease contexts such as anemia or hemorrhagic shock, where both oxygen delivery and CO_2_ removal are impaired. Scaling up the synthesis and integrating stealth or antioxidant coatings may further strengthen the translational potential of these nanocarriers.

Scaling up the synthesis also presents hurdles. As with liposomes and polymeric nanoparticles, reproducing laboratory conditions at industrial scale introduces risks of variability, aggregation, and inconsistent particle size, while MOFs additionally face challenges such as the need for high-purity precursors, removal of residual solvents, and control over metal ion release. Addressing these bottlenecks will likely require robust, continuous-flow or microreactor-based methods capable of delivering reproducible, safe, and cost-effective large-scale production.

Altogether, despite these challenges, this study establishes Hb@ZIF-8/PEG NPs as a versatile and promising platform. With further optimization in stability, scalability, and *in vivo* validation, these nanocarriers have strong potential to evolve into next-generation multifunctional artificial RBCs capable of addressing critical clinical needs.

## CRediT authorship contribution statement

**Ana María Pablo-Sainz-Ezquerra:** Writing – review & editing, Writing – original draft, Methodology, Investigation. **Marta Rubio-Huertas:** Writing – review & editing, Methodology, Investigation. **Ege Tini Tunca:** Writing – review & editing, Methodology, Investigation. **Peter Waaben Thulstrup:** Writing – review & editing, Supervision, Methodology, Investigation. **Leticia Hosta-Rigau:** Writing – review & editing, Writing – original draft, Supervision, Resources, Project administration, Funding acquisition, Conceptualization.

## Declaration of generative AI and AI-assisted technologies in the writing process

During the preparation of this work the authors used Chat GPT in order to improve the readability of the manuscript. After using this tool/service, the authors reviewed and edited the content as needed and take full responsibility for the content of the publication.

## Funding sources

This research was funded by the 10.13039/501100000781European Research Council under the European Union's 10.13039/501100007601Horizon 2020 Research and Innovation Program (Grant No. 101002060).

## Declaration of competing interest

The authors declare that they have no known competing financial interests or personal relationships that could have appeared to influence the work reported in this paper.

## Data Availability

Data will be made available on request.
