# Supplementary material for: Dual-function hemoglobin-encapsulating ZIF-8 nanoparticles: Oxygen transport capability and carbonic anhydrase-like activity
Source: Mater Today Bio. 2025 Oct 10;35:102406. doi: 10.1016/j.mtbio.2025.102406 (PMC12554216; doi:10.1016/j.mtbio.2025.102406)
Supplement: Multimedia component 1 [file mmc1.docx]

Supporting Information

Dual-Function Hemoglobin-Encapsulating ZIF-8 Nanoparticles: Oxygen Transport Capability and Carbonic Anhydrase-like Activity

Ana María Pablo‑Sainz‑Ezquerra,^a^ Marta Rubio‑Huertas,^a^ Ege Tini Tunca,^a^ Peter Waaben Thulstrup,^b^ and Leticia Hosta‑Rigau ^a,*^

^a^ Department of Health Technology, Technical University of Denmark, Nils Koppels Allé, Building 423, 2800 Kgs. Lyngby, Denmark.

^c^ Department of Chemistry, University of Copenhagen, Universitetsparken 5, 2100 Copenhagen, Denmark.

* E‑mail: [leri@dtu.dk](mailto:leri@dtu.dk)

**Table S1.** Statistical analysis of Michaelis-Menten kinetics using ANOVA F-tests. Maximum velocity (V_max_) and Michaelis-Menten constant (K_m_), their standard error (SE), adjusted R-squared (R^2^) and F-values (F(df1,df2)) and associated p-values associated with the F-statistics (p(F)) for each sample and replicate are reported.

| Sample | Replicate | Parameter | Value | SE | R^2^ | F(df1,df2) | p(F) |
| --- | --- | --- | --- | --- | --- | --- | --- |
| ZIF-8 NPs | 1 | V_max_ | 3.15E-07 | 2.20E-08 | 0.9983 | 3138.0 | <0.0001 |
|  |  | K_m_ | 0.00661 | 6.56E-04 |  |  |  |
|  | 2 | V_max_ | 2.86E-07 | 2.47E-08 | 0.9977 | 2079.9 | <0.0001 |
|  |  | K_m_ | 0.00631 | 7.41E-04 |  |  |  |
| ZIF-8/PEG NPs | 1 | V_max_ | 6.24E-08 | 1.17E-08 | 0.9907 | 404.0 | <0.0001 |
|  |  | K_m_ | 0.00186 | 4.95E-04 |  |  |  |
|  | 2 | V_max_ | 8.10E-08 | 2.49E-08 | 0.9733 | 176.3 | 1.26E-04 |
|  |  | K_m_ | 0.00245 | 0.0011 |  |  |  |
| Hb0.6@ZIF-8/PEG NPs | 1 | V_max_ | 2.05E-07 | 2.81E-08 | 0.9908 | 560.7 | <0.0001 |
|  |  | K_m_ | 0.00643 | 0.00125 |  |  |  |
|  | 2 | V_max_ | 1.79E-07 | 6.29E-08 | 0.9420 | 91.8 | 1.14E-04 |
|  |  | K_m_ | 0.00597 | 0.00268 |  |  |  |
| Hb3@ZIF-8/PEG NPs | 1 | V_max_ | 4.32E-08 | 2.61E-09 | 0.9913 | 732.4 | <0.0001 |
|  |  | K_m_ | 0.00123 | 1.57E-04 |  |  |  |
|  | 2 | V_max_ | 4.14E-08 | 4.69E-09 | 0.9618 | 163.2 | <0.0001 |
|  |  | K_m_ | 8.99E-04 | 2.18E-04 |  |  |  |
| Hb6@ZIF-8/PEG NPs | 1 | V_max_ | 3.12E-08 | 9.15E-10 | 0.9979 | 2897.2 | <0.0001 |
|  |  | K_m_ | 0.00161 | 1.16E-04 |  |  |  |
|  | 2 | V_max_ | 3.36E-08 | 4.62E-10 | 0.9996 | 21787.8 | <0.0001 |
|  |  | K_m_ | 9.76E-04 | 4.10E-05 |  |  |  |
| Hb13@ZIF-8/PEG NPs | 1 | V_max_ | 8.07E-09 | 3.19E-09 | 0.9433 | 138.7 | 2.02E-04 |
|  |  | K_m_ | 0.00121 | 7.01E-04 |  |  |  |
|  | 2 | V_max_ | 6.34E-09 | 1.88E-09 | 0.9386 | 83.0 | 5.54E-04 |
|  |  | K_m_ | 7.61E-04 | 4.49E-04 |  |  |  |
| Hb25@ZIF-8/PEG NPs | 1 | V_max_ | 6.66E-09 | 1.76E-09 | 0.9433 | 145.6 | 0.00103 |
|  |  | K_m_ | 9.14E-04 | 3.88E-04 |  |  |  |
|  | 2 | V_max_ | 8.00E-09 | 1.88E-09 | 0.9211 | 87.7 | 1.28E-04 |
|  |  | K_m_ | 0.00148 | 5.41E-04 |  |  |  |
| CA | 1 | V_max_ | 1.64E-06 | 5.60E-07 | 0.9722 | 140.6 | <0.0001 |
|  |  | K_m_ | 0.00329 | 0.00129 |  |  |  |
|  | 2 | V_max_ | 3.64E-06 | 6.56E-07 | 0.9960 | 750.6 | <0.0001 |
|  |  | K_m_ | 0.00554 | 0.00115 |  |  |  |

**Synthesis scheme of ZIF-8 NPs, ZIF-8/PEG NPs and Hbx@ZIF-8/PEG NPs (x=0.6, 3, 6, 13, 25).**

**
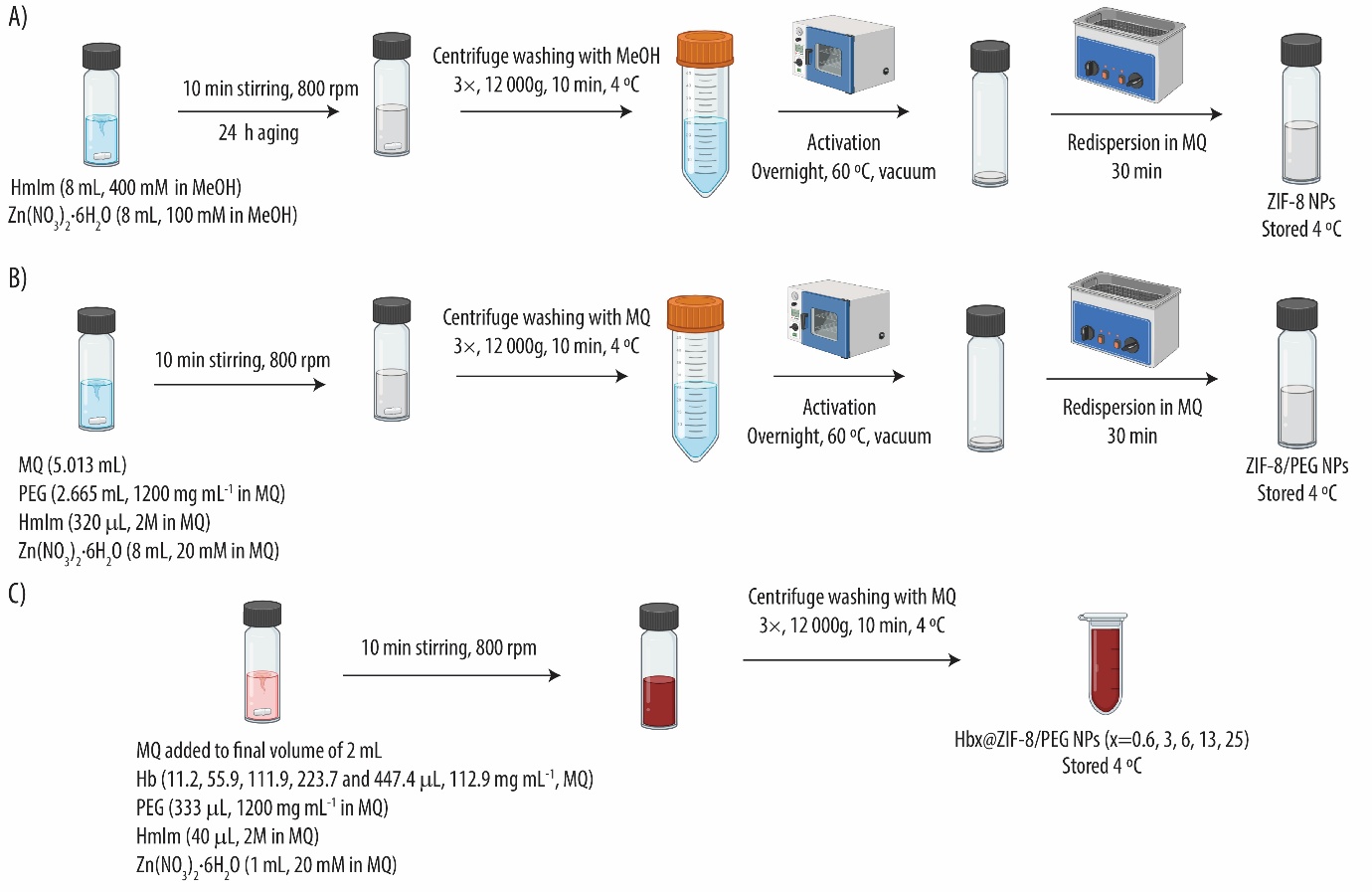
**

**Figure S1.** Schematic illustration of the synthesis routes: (A) ZIF-8 nanoparticles (NPs) in methanol (MeOH), (B) ZIF-8/PEG NPs in Milli-Q water (MQ), and (C) hemoglobin-loaded ZIF-8/PEG NPs (Hbx@ZIF-8/PEG, x = 0.6, 3, 6, 13, 25) in MQ. Reactants were added sequentially in the order shown (top to bottom). All syntheses were performed at room temperature (RT). Abbreviations: 2-methylimidazole (HmIm), polyethylene glycol (PEG), hemoglobin (Hb).

**Hemoglobin activity towards the conversion of *p*‑NPA into *p*‑NP.**

**
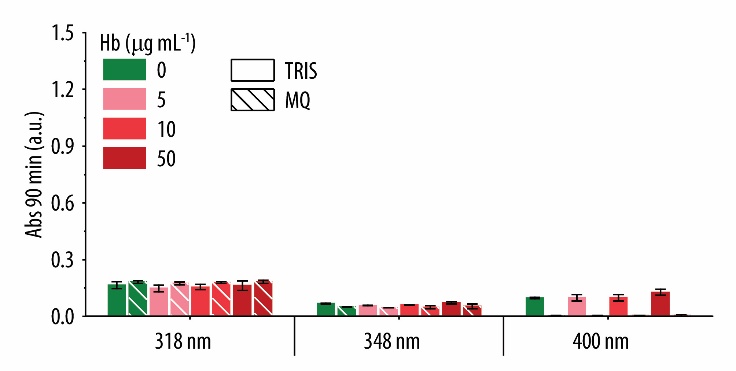
**

**Figure S2.** Absorbance (Abs) signals at 318, 348 and 400 nm after 90 min of reaction of different Hb concentrations with *p*‑nitrophenyl acetate (*p*‑NPA) (0.2 mM) in 20 mM TRIS buffer at pH 7.4 (solid bars) and in Milli-Q (MQ) water (stripped bars) at room temperature to produce *p*‑nitrophenol (*p*‑NP) and acetic acid.


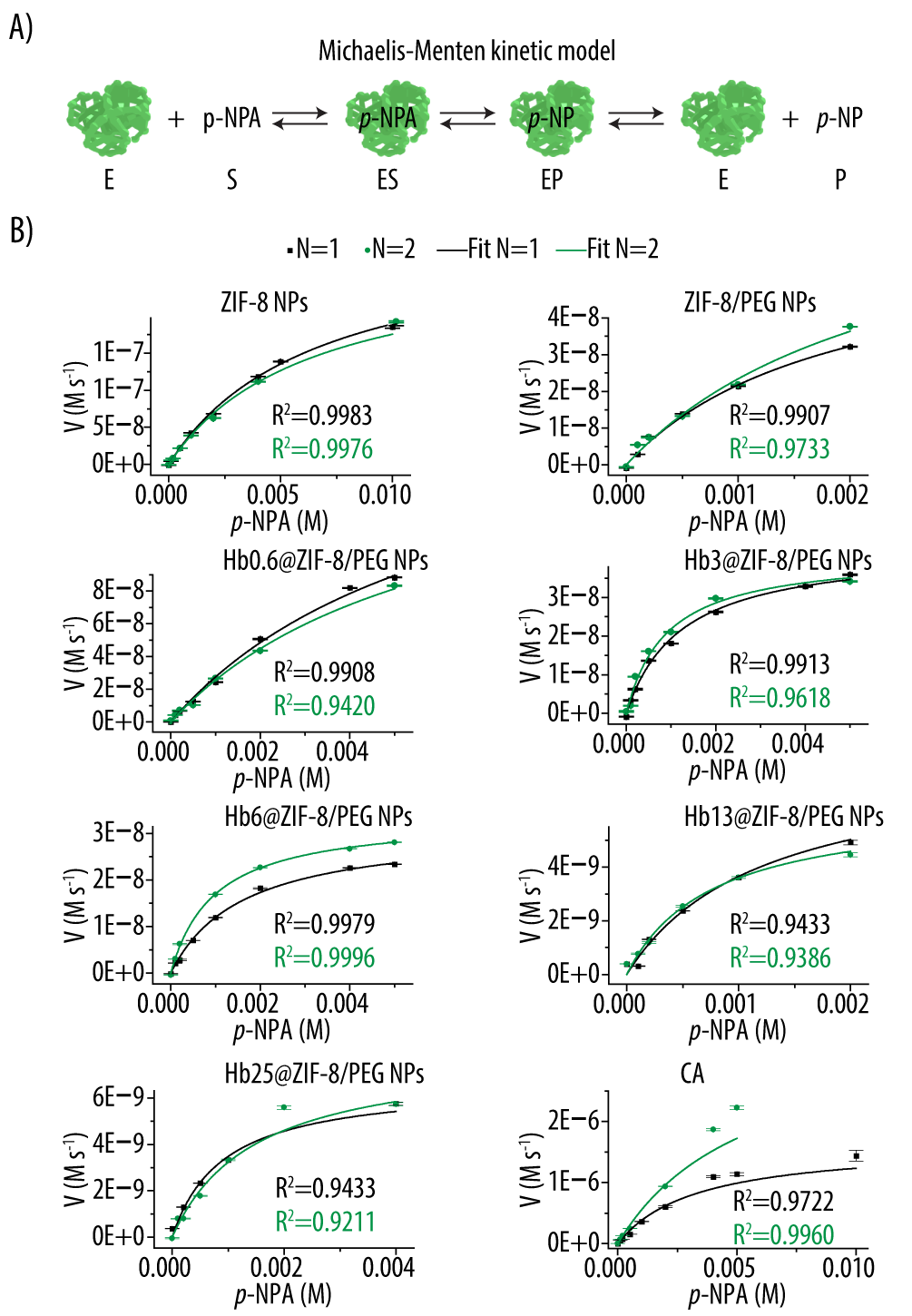


**Figure S3.** **A)** Michaelis‑Menten model illustrating the mechanism of an enzyme‑catalyzed reaction, including enzyme (E), substrate (S), enzyme‑substrate complex (ES), enzyme‑product complex (EP), and product (P). **B)** Michaelis‑Menten fitted curves of ZIF‑8, ZIF‑8/PEG, and Hbx@ZIF‑8/PEG NPs (x = 0.6, 3, 6, 13, 25) at 100 μg mL^−1^ incubated with *p*‑nitrophenyl acetate (*p*‑NPA) all in Milli-Q water (MQ), and room temperature (RT). *p*‑nitrophenyl acetate (*p*‑NPA). As a positive control, carbonic anhydrase (CA) at 50 μg mL^−1^ with *p*‑NPA all in TRIS buffer (20 mM, pH 7.4) and RT was considered. x = 0.6, 3, 6, 13, 25 indicates the concentration of Hb in mg mL^−1^ used for the NPs assembly.

**Hb, Hbx@ZIF‑8/PEG NPs and ZIF‑8/PEG NPs UV‑Vis spectra before N_2_ and air purging.**


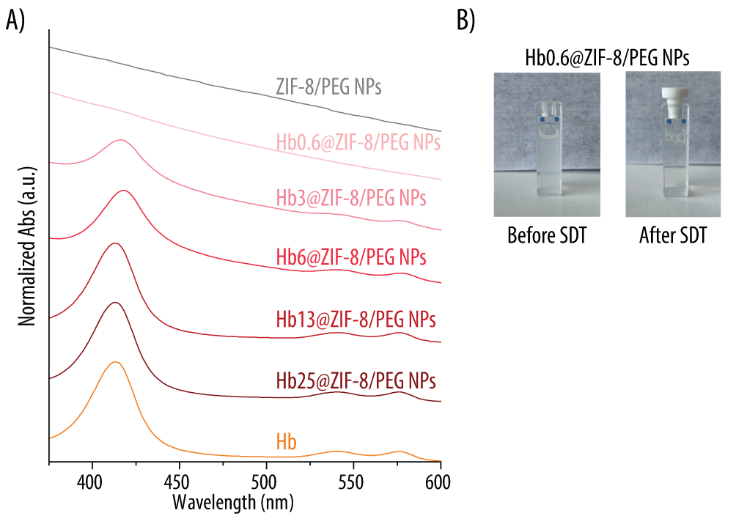


**Figure S4.** **A)** UV‑Vis spectra of Hb, Hbx@ZIF‑8/PEG NPs (x = 0.6, 3, 6, 13, 25) and ZIF‑8/PEG NPs in Milli‑Q water (MQ). **B)** Photographs of Hb0.6@ZIF‑8/PEG NPs sample before and after the addition of sodium dithionite (SDT).

**Molecular structure of fluorescein diacetate and calcein‑AM.**


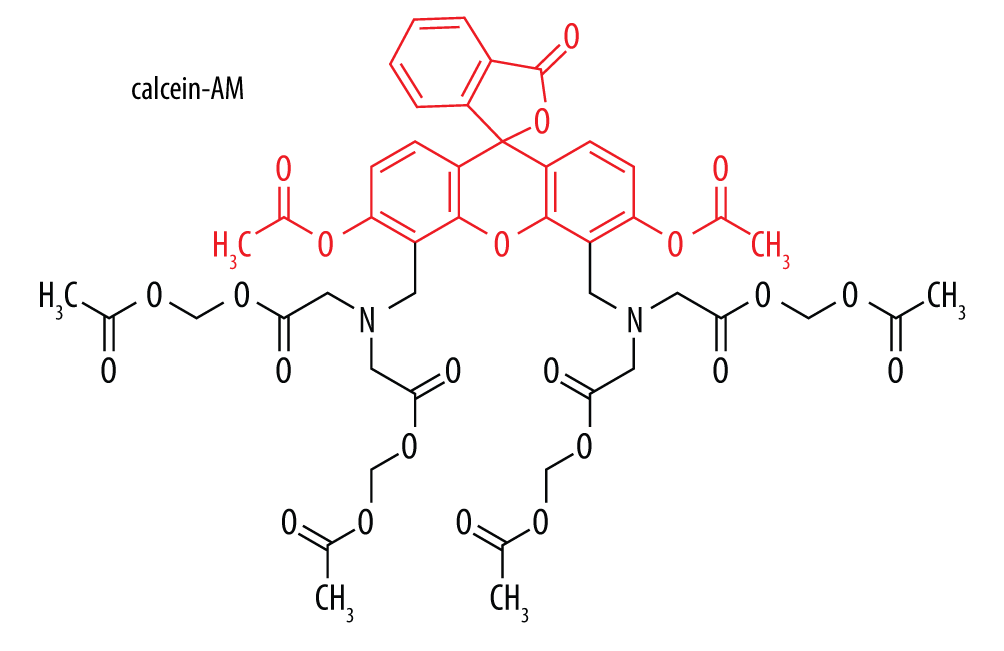


**Figure S5.** Molecular structure of calcein‑acetoxymethyl (calcein‑AM).

**Calcein‑AM fluorescence produced by Hb.**


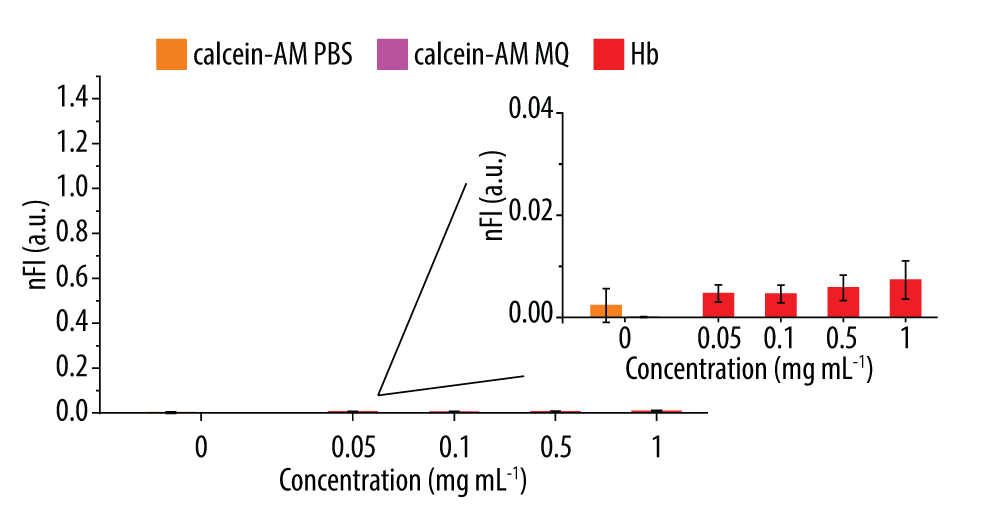


**Figure S6.** Fluorescent activity towards calcein‑acetoxymethyl (calcein‑AM) of hemoglobin (red) and the controls in PBS (orange) and Milli‑Q (MQ) water (purple).
